# Valsartan Improves Adipose Tissue Function in Humans with Impaired Glucose Metabolism: A Randomized Placebo-Controlled Double-Blind Trial

**DOI:** 10.1371/journal.pone.0039930

**Published:** 2012-06-29

**Authors:** Gijs H. Goossens, Chantalle C. M. Moors, Nynke J. van der Zijl, Nicolas Venteclef, Rohia Alili, Johan W. E. Jocken, Yvonne Essers, Jack P. Cleutjens, Karine Clément, Michaela Diamant, Ellen E. Blaak

**Affiliations:** 1 Department of Human Biology, NUTRIM School for Nutrition, Toxicology and Metabolism, Maastricht University Medical Center, Maastricht, The Netherlands; 2 Diabetes Center, Department of Internal Medicine, VU University Medical Center, Amsterdam, The Netherlands; 3 INSERM, Nutriomique U872 (Eq 7), University Pierre et Marie Curie-Paris 6, Cordelier Research Center, Human Nutrition Research Center; Assistance Publique-Hôpitaux de Paris, Pitié-Salpêtrière hospital, Paris, France; 4 Department of Pathology, Cardiovascular Research Institute Maastricht, Maastricht University Medical Center, Maastricht, The Netherlands; Catholic University Medical School, Italy

## Abstract

**Background:**

Blockade of the renin-angiotensin system (RAS) reduces the incidence of type 2 diabetes mellitus. In rodents, it has been demonstrated that RAS blockade improved adipose tissue (AT) function and glucose homeostasis. However, the effects of long-term RAS blockade on AT function have not been investigated in humans. Therefore, we examined whether 26-wks treatment with the angiotensin II type 1 receptor blocker valsartan affects AT function in humans with impaired glucose metabolism (IGM).

**Methodology/Principal Findings:**

We performed a randomized, double-blind, placebo-controlled parallel-group study, in which 38 subjects with IGM were treated with valsartan (VAL, 320 mg/d) or placebo (PLB) for 26 weeks. Before and after treatment, an abdominal subcutaneous AT biopsy was collected for measurement of adipocyte size and AT gene/protein expression of angiogenesis/capillarization, adipogenesis, lipolytic and inflammatory cell markers. Furthermore, we evaluated fasting and postprandial AT blood flow (ATBF) (^133^Xe wash-out), systemic inflammation and insulin sensitivity (hyperinsulinemic-euglycemic clamp). VAL treatment markedly reduced adipocyte size (*P*<0.001), with a shift toward a higher proportion of small adipocytes. In addition, fasting (*P* = 0.043) and postprandial ATBF (*P* = 0.049) were increased, whereas gene expression of angiogenesis/capillarization, adipogenesis and macrophage infiltration markers in AT was significantly decreased after VAL compared with PLB treatment. Interestingly, the change in adipocyte size was associated with alterations in insulin sensitivity and reduced AT gene expression of macrophage infiltration markers. VAL did not alter plasma monocyte-chemoattractant protein (MCP)-1, TNF-α, adiponectin and leptin concentrations.

**Conclusions/Significance:**

26-wks VAL treatment markedly reduced abdominal subcutaneous adipocyte size and AT macrophage infiltration markers, and increased ATBF in IGM subjects. The VAL-induced decrease in adipocyte size was associated with reduced expression of macrophage infiltration markers in AT. Our findings suggest that interventions targeting the RAS may improve AT function, thereby contributing to a reduced risk of developing cardiovascular disease and type 2 diabetes.

**Trial Registration:**

Trialregister.nl NTR721 (ISRCTN Registry: ISRCTN42786336)

## Introduction

Multiple lines of evidence suggest that increased activation of the renin-angiotensin system (RAS) is involved in the development of type 2 diabetes mellitus [1]–[4]. A meta-analysis of comparative outcome trials has demonstrated that RAS blockade reduced the incidence of new-onset type 2 diabetes by 22% in high-risk subjects [5]. More recently, the prospective NAVIGATOR trial has shown that treatment with the angiotensin (Ang) II type 1 receptor blocker (ARB) valsartan, in addition to lifestyle modification, reduced type 2 diabetes incidence by 14% in subjects with impaired glucose tolerance (IGT) [6]. Thus, these findings corroborate the assertion that RAS blockade is protective against the development of type 2 diabetes in humans.

The beneficial effects of RAS blockade in the prevention of type 2 diabetes have been explained by improved insulin sensitivity and insulin secretion [1]–[3]. Studies that have investigated the effect of short-term RAS blockade on insulin sensitivity, however, report conflicting results [7], and the underlying mechanisms are not yet fully understood. RAS components have been identified in a variety of tissues, including adipose tissue (AT), and it has been demonstrated that increased activation of the RAS is linked to insulin resistance [1]. Converging evidences suggest that several aspects of AT dysfunction, including macrophage infiltration, inflammation and impaired AT blood flow (ATBF), contribute to insulin resistance [8]. Thus, it is tempting to postulate that RAS blockade may improve AT function, thereby increasing insulin sensitivity. Indeed, RAS blockade decreased adipocyte size and AT gene expression of inflammatory markers, and improved glucose homeostasis in rodents [9]–[14]. However, it is presently unknown whether long-term RAS blockade evokes beneficial effects on AT function in humans.

The objective of the present study was to investigate whether long-term RAS blockade improves AT function in subjects with impaired glucose metabolism (IGM). We have recently conducted a randomized placebo-controlled trial and demonstrated that 26-wks VAL treatment improved both insulin sensitivity and glucose-stimulated insulin secretion in subjects with IGM [15]. To elucidate the underlying mechanisms for improved insulin sensitivity, the present study examined the effects of 26-wks RAS blockade on several factors associated with AT function, including adipocyte size, ATBF, gene expression of angiogenesis/capillarization, adipogenesis, lipolytic and inflammatory cell markers in AT, and systemic inflammation.

## Methods

### Ethics Statement

The present study was conducted within the framework of the PRESERVE study, a randomized double-blind, placebo-controlled, parallel-group study carried out in two different centers in The Netherlands (Maastricht (*n* = 38), Amsterdam (*n* = 41)) [15]. The original protocol for this trial and supporting CONSORT checklist are available as supporting information; see Checklist S1 and Protocol S1. Just before the start of the trial, we decided not to initiate the rosiglitazone arm because of the reported potential cardiovascular risks associated with rosiglitazone [16]. The present sub-study was performed at Maastricht University Medical Center. All clinical investigations conformed to the standards set by the *Declaration of Helsinki*, the Medical-Ethical Committee of Maastricht University Medical Center approved the study protocol, including the present sub-study, and subjects gave their written informed consent before participation.

### Subjects

**Figure 1:**
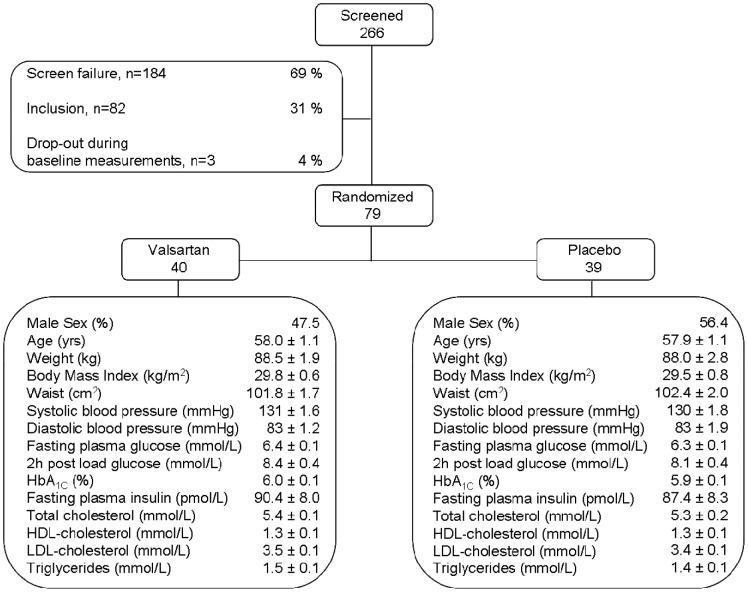
Enrollment flow chart.

Figure 1 describes the flow of participants through the main trial [15]. Eligible participants were assigned to valsartan or placebo according to a computer-generated, randomization plan (block size, n = 4). The local pharmacy (Maastricht University Medical Center) generated the random allocation sequence and assigned participants to interventions.

In the present sub-study, thirty-eight subjects with impaired fasting glucose (IFG; fasting plasma glucose ≥6.1 and <7.0 mmol/l or FPG ≥5.6 and <7.0 mmol/l with family history (first-degree relative) of type 2 diabetes), and/or IGT (2 h plasma glucose concentration ≥7.8–11.1 mmol/l) (isolated IFG, *n* = 19; isolated IGT, *n* = 9; combined IFG/IGT, *n* = 10) were randomly assigned to VAL (n = 19) or PLB (n = 19) treatment. Subjects received 160 mg VAL or PLB once daily (q.d.) for 2 weeks. Thereafter, the dosage was doubled to 320 mg q.d. VAL or PLB for the subsequent 24 wks. Study medication was taken in the evening throughout the treatment period. Before and after 26-wks treatment, a hyperinsulinemic-euglycemic clamp, OGTT, and postprandial study (measurement of fasting and postprandial ATBF) were performed, and an AT biopsy was collected. All baseline procedures were randomized and completed within 5 weeks after inclusion, with at least 1 week between measurements, after an overnight fast. We were not able to obtain AT biopsies of sufficient quality for mRNA expression (low RIN-value) in 6 VAL-treated subjects, were not able to quantify adipocyte size due to tissue damage in 7 subjects (3 PLB and 4 VAL-treated subjects), and were unable to perform ATBF measurements in 8 subjects (5 PLB and 3 VAL-treated subjects) either before or after treatment. One data-point for insulin sensitivity (1 VAL subject) was considered an outlier in the statistical analysis.

Exclusion criteria were diabetes mellitus, cardiovascular disease, cancer, lung disease, abnormal liver (alanine aminotransferase (ALAT)>35 U/l and gamma-glutamyl transferase (GGT)>35 U/l) and renal function (creatinine>110 µmol/l) tests, intentions to lose weight or follow a hypocaloric diet and alcohol or drug abuse. Subjects were only allowed to use statins (*n* = 1). Subjects with a blood pressure >140/90 mmHg at screening were treated with 5 mg amlodipine. If blood pressure >140/90 mmHg persisted, the amlodipine dosage was increased to 10 mg, followed by addition of hydrochlorothiazide 12.5 mg and/or carvedilol 25 mg if needed. Individuals with a blood pressure <140/90 mmHg entered the study. Fourteen subjects required amlodipine (VAL group, 6 subjects; PLB group, 8 subjects), and in seven of these subjects hydrocholothiazide was added (VAL group, 2 subjects; PLB group, 5 subjects) to control blood pressure.

### Adipose Tissue Biopsy

An abdominal subcutaneous AT biopsy (∼1 g) was collected 6–8 cm lateral from the umbilicus under local anesthesia (2% lidocaine) by needle biopsy after an overnight fast. AT was washed with sterile saline and processed within 5 min. A small part of the AT was fixed overnight in 4% paraformaldehyde and embedded in paraffin, whereas the other part was snap frozen in liquid nitrogen and stored at -80°C.

#### Adipocyte size

Histological sections (8 µm) were cut from paraffin-embedded tissue, mounted on microscope glass slides and dried overnight in an incubator at 37°C. Sections were stained with haematoxylin and eosin. Digital images were captured using a Leica DFC320 digital camera (Leica DM3000 microscope, Leica, Rijswijk, The Netherlands) at 20× magnification. Computerized morphometric analysis (Leica QWin V3, Cambridge, UK) of individual adipocytes (>400 adipocytes/sample) was performed in a blinded fashion, with a coefficient of variation <5%.

#### Quantitative RT-PCR

Total RNA was extracted from AT (∼500 mg) using Trizol chloroform extraction (Invitrogen, Cergy Pontoise, France) for measurement of gene expression of angiogenesis/capillarization, adipogenesis, lipolytic and inflammatory cell markers. SYBR-Green based real-time PCRs were performed as one-step reactions on the StepOne real-time PCR system (Applied Biosystems, Foster City, CA). Results were normalized to 18 S ribosomal RNA.

#### Western blot analysis

AT (∼200 mg) was ground to fine powder under liquid nitrogen and homogenized in 200 µl of ice-cold buffer [17]. The homogenate was vortexed, centrifuged (20,000 g, 30 min, 4°C), and supernatant was collected and stored at -80°C. The protein concentration was determined by the Bradford-based protein assay (catalog no. 500-0006; Bio-Rad). Next, protein expression of ATGL and HSL was measured, as described previously [17]. CGI-58 was detected using a rabbit polyclonal antibody raised against human CGI-58 (Novus Biologicals, NB110-41576). The G0S2 antibody [18] was a kind gift from Dr. Sander Kersten (Wageningen University, The Netherlands).

### Fasting and Postprandial ATBF

ATBF was continuously measured at baseline and for 4 h after consumption of a standardized high-fat mixed meal (consisting of 61E% fat (35.5E% saturated fatty acids (FAs), 18.8E% monounsaturated FAs and 1.7E% polyunsaturated FAs), 33E% carbohydrate and 6E% protein, containing 2.6 MJ), as previously described [19], [20]. Briefly, ^133^Xe (∼1 MBq) was injected para-umbilically into the adipose tissue, approximately 10 mm deep. A CsI crystal detector (Oakfield Instruments, Eynsham, UK) was placed over the exact site of injection and taped firmly in place to monitor the mono-exponential decay of radioactivity in the adipose tissue. This γ-counter probe collected continuous 20 s readings.

### Hyperinsulinemic-euglycemic Clamp

A hyperinsulinemic-euglycemic clamp was performed to assess insulin sensitivity [21]. Briefly, a cannula was inserted into a superficial dorsal hand vein for sampling of arterialized blood and in an antecubital vein of the contralateral forearm for insulin (40 mU/m^2^/min, Actrapid, Novo Nordisk Farma BV, Alphen aan den Rijn, The Netherlands) and glucose infusion. Blood glucose concentration was measured every 5 min (EML 105, Radiometer, Copenhagen, Denmark) and euglycemia was maintained at 5.0 mmol/l (variable 20% glucose infusion). The mean glucose infusion rate (M-value) during steady-state (last 30 min of the clamp) was used to assess insulin sensitivity.

### Biochemical Analyses

Blood samples were collected into ice-chilled EDTA-tubes and centrifuged at 1000 g, 4°C for 10 min. Plasma was immediately frozen in liquid nitrogen and stored at -80°C until analysis. Plasma glucose concentrations were determined using a hexokinase method (Gluco-quant, Roche Diagnostics, Mannheim, Germany). HbA1c was measured by cation exchange chromatography (Menarini Diagnostics, Florence, Italy). Plasma insulin concentrations were quantified using an immunometric assay (Advia, Siemens Medical Solutions Diagnostics, IL). Serum monocyte chemoattractant protein-1 (MCP-1), tumour necrosis factor-α (TNF-α) and leptin concentrations were measured using the Bioplex Protein Array System (Bio-Rad Laboratories, Hercules, CA) by fluorescent conjugated monoclonal antibodies. Adiponectin concentration was determined using ELISA (Quantikine, R&D Systems, Abingdon, UK).

### Statistical Analysis

Baseline comparisons between treatment groups were analyzed using Student’s unpaired *t* test. Treatment effects were assessed by repeated-measures ANOVA, using time as within-subject factor and treatment as between-subject factor, with adjustment for gender and glucometabolic status (IFG, IGT or combined IFG/IGT). Univariate correlations were used to examine associations between parameters. Since adjustment for gender and glucometabolic status did not affect the results, unadjusted values were used. All variables were checked for normal distribution, and variables with a skewed distribution were ln-transformed to satisfy conditions of normality. Data are presented as means±SEM, or as medians (interquartile range) in case of non-normal distribution. Calculations were done using SPSS 15.0 for Windows (Chicago, IL, USA). *P*<0.05 was considered to be statistically significant.

## Results

All individuals randomized completed the present study. Subject characteristics are summarized in Table 1. Before the start of treatment, age, BMI, body fat percentage, waist and hip circumferences, blood pressure, lipid profile, plasma glucose and insulin concentrations, and insulin sensitivity were comparable between subjects randomized to VAL or PLB. The study medication was well-tolerated and no serious adverse events were reported.

**Table 1:** Subject characteristics before and after 26-wks treatment with VAL or PLB.

	Valsartan (*n* = 19)		Placebo (*n* = 19)		
	Baseline	26 wks	Baseline	26 wks	*P*-value*
Sex (male/female)	7/12	–	11/8	–	–
Age (yr)	59.4±1.5	–	59.2±1.2	–	*N.S.*
Weight (kg)	86.2±3.0	86.6±3.3	90.2±4.0	90.1±3.9	*N.S.*
BMI (kg/m^2^)	30.6±1.0	30.7±1.1	30.9±1.2	30.9±1.2	*N.S.*
Total body fat (%)	34.6±1.9	34.3±1.9	31.8±1.5	32.3±1.6	0.073
Trunk fat (%)	35.3±1.9	35.5±1.9	33.7±1.5	34.3±1.5	*N.S.*
Waist (cm)	101.1±2.4	102.8±3.1	104.7±3.1	105.3±2.9	*N.S.*
Hip (cm)	105.5±2.2	106.6±2.5	103.0±2.0	105.6±2.3	*N.S.*
WHR	0.96±0.02^#^	0.96±0.02	1.02±0.02	1.00±0.02	*N.S.*
SBP (mmHg)	127.0±2.1	111.5±2.4	130.2±3.0	124.5±2.8	0.004
DBP (mmHg)	79.5±1.6	70.8±1.1	80.1±2.0	78.6±1.9	0.009
Fasting glucose (mmol/l)	6.4±0.1	6.2±0.2	6.2±0.1	5.9±0.1	*N.S.*
2h-glucose (mmol/l)	7.5±0.6	7.7±0.6	7.6±0.6	8.5±0.6	*N.S.*
Fasting insulin (pmol/l)	103.0±20.6	104.0±14.7	100.7±10.9	100.3±8.9	*N.S*.
HbA1C (%) (mmol/mol)	6.1±0.1 (42.9±1.3)	6.0±0.1 (41.6±1.2)	6.0±0.1 (41.8±1.0)	6.0±0.1 (42.3±1.2)	*N.S.*
M-value (mg·min^−1^·kg^−1^)	3.4±0.4	3.6±0.4	3.2±0.4	3.1±0.3	*N.S.*
Fasting TAG (mmol/l)	1.23±0.08	1.48±0.10	1.39±0.15	1.69±0.23	*N.S.*
Fasting NEFA (µmol/l)	570.0±43.9	520.6±46.1	577.9±40.2	567.5±72.3	*N.S.*

BMI, body mass index; HbA1C, glycated haemoglobin; NEFA, non-esterified fatty acid; SBP, systolic blood pressure; DBP, diastolic blood pressure; TAG, triacylglycerol; WHR, waist-to-hip ratio; *N.S.*, not significant. ^*^VAL *vs.* PLB treatment assessed by repeated-measures ANOVA. ^#^ *P*<0.05 *vs*. PLB. Values are means±SEM.

### Blood Pressure

At baseline, both systolic and diastolic blood pressure was comparable between treatment groups (Table 1). VAL treatment decreased systolic (VAL: −15.7±3.2 *vs.* PLB: −4.3±1.7 mmHg, *P* = 0.004) and diastolic (VAL: −8.7±2.1 *vs.* PLB: −2.0±1.4 mmHg, *P* = 0.009) blood pressure compared with PLB (Table 1).

### Adipocyte Size

**Figure 2:**
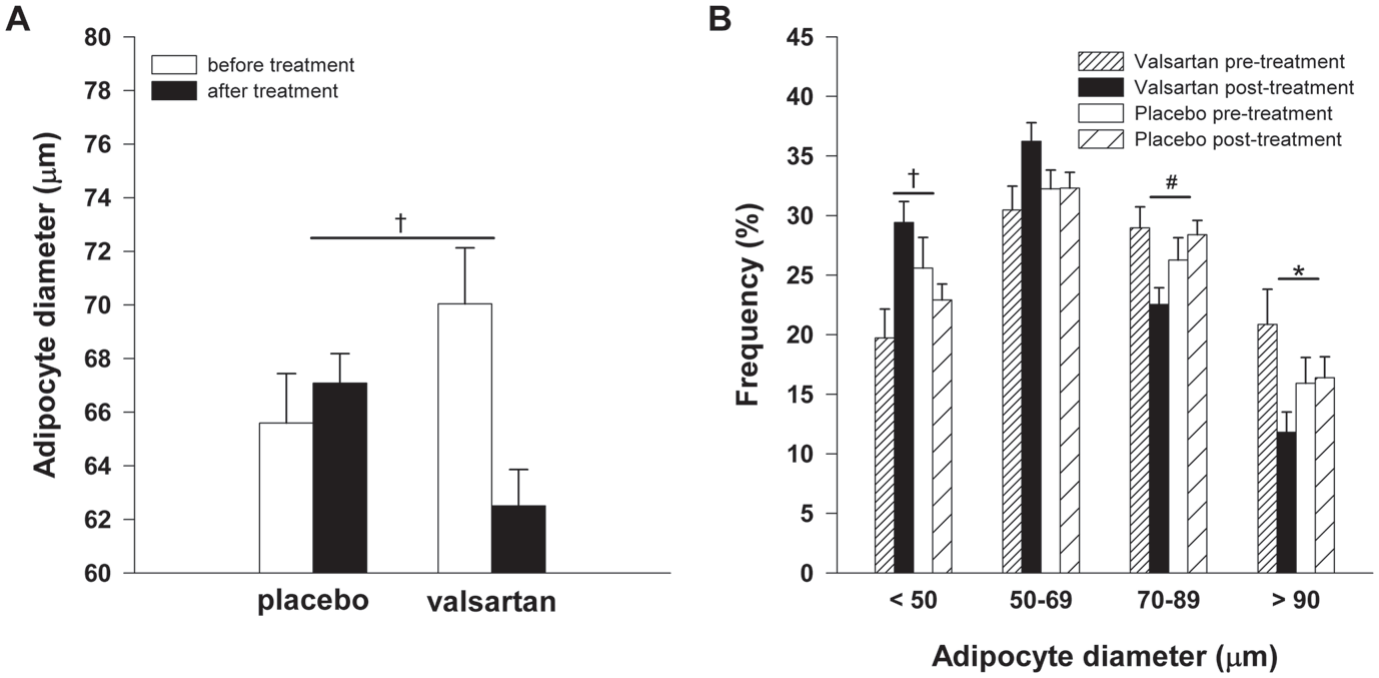
Mean adipocyte diameter and adipocyte size distribution VAL treatment (*n* = 15) significantly reduced (**A**) adipocyte size compared with PLB (*n* = 16), with (**B**) a shift toward a higher proportion of small adipocytes. Values are means±SEM. *P<0.05, ^#^ *P*<0.01, ^†^ *P*<0.001 VAL vs. PLB.

At baseline, abdominal subcutaneous adipocyte size was comparable between treatment groups (VAL: 70.0±2.1 *vs.* PLB: 65.6±1.8 µm, *P* = 0.121). VAL treatment markedly reduced mean adipocyte size compared with PLB (*P*<0.001, Figure 2A), with a shift toward a higher proportion of small adipocytes (Figure 2B). Noteworthy, the reduction in adipocyte size was a very consistent finding, since this was observed in all subjects treated with VAL.

AT gene expression of peroxisome proliferator-activated receptor (PPAR)γ (*P* = 0.061), adipocyte fatty acid binding protein (aP2) (*P* = 0.016) and CCAAT/enhancer binding protein (C/EBP)α (*P* = 0.006), which are key regulatory factors in adipogenesis and lipogenesis [22], were decreased after VAL treatment compared with PLB (Table 2).

**Table 2:** Abdominal subcutaneous AT gene expression before and after 26-wks treatment with VAL or PLB.

	Valsartan (*n* = 19)		Placebo (*n* = 13)		
	Baseline	26 wks	Baseline	26 wks	P*
**Adipogenesis**					
PPARγ mRNA	1.25 (0.64–1.72)	0.94 (0.55–1.15)	0.83 (0.42–1.06)	1.51 (0.53–1.69)	0.061
aP2 mRNA	0.86 (0.38–1.31)	0.45 (0.27–0.62)	0.65 (0.37–0.90)	0.74 (0.39–1.07)	0.016
C/EBPα mRNA	1.12 (0.82–0.87)	0.94 (0.60–1.07)	1.13 (0.71–1.54)	1.27 (0.90–1.83)	0.006
**Capillarization**					
VEGF mRNA	0.58 (0.41–0.94)	0.54 (0.31–0.66)	0.50 (0.23–0.76)	0.63 (0.32–0.72)	0.051
CD34 mRNA	0.78 (0.60–1.18)	0.65 (0.49–1.16)	0.73 (0.34–1.02)	1.03 (0.45–1.44)	0.037
ANG mRNA	0.58 (0.41–0.68)	0.50 (0.21–0.66)	0.39 (0.32–0.67)	0.46 (0.38–0.77)	0.028
**Inflammation**					
CD68 mRNA	1.97 (0.80–2.52)	0.94 (0.63–1.74)	0.79 (0.64–1.58)	1.78 (0.98–2.93)	0.014
CD163 mRNA	1.21 (0.87–1.99)	0.80 (0.56–1.20)	1.00 (0.91–1.59)	1.41 (1.05–1.82)	0.023
CD206 mRNA	1.17 (0.85–1.73)	0.90 (0.53–1.45)	1.03 (0.63–1.31)	1.35 (0.81–2.38)	0.004
CD11b mRNA	1.58 (0.78–2.31)	1.54 (0.61–2.18)	1.11 (0.77–2.05)	1.94 (1.02–2.89)	0.364
CTSS mRNA	1.01 (0.48–1.37)	0.79 (0.35–1.07)	0.57 (0.40–0.82)	0.79 (0.57–1.40)	0.014
**Lipolysis**					
ATGL mRNA	0.74 (0.37–1.11)	0.39 (0.28–0.74)	0.65 (0.48–1.04)	0.68 (0.50–1.27)	0.083
CGI-58 mRNA	0.52 (0.35–0.78)	0.34 (0.20–0.49)	0.35 (0.23–0.88)	0.52 (0.25–0.86)	0.090
HSL mRNA	2.10 (1.42–3.14)	1.60 (0.96–2.46)	3.10 (1.54–4.42)	2.48 (1.64–3.85)	0.710
G0S2 mRNA	0.87 (0.56–1.39)	0.59 (0.38–1.08)	0.78 (0.54–1.22)	0.99 (0.48–1.76)	0.092

PPARγ, peroxisome proliferator-activated receptor γ; aP2, adipocyte fatty acid binding protein; C/EBPα, CCAAT/enhancer binding protein α; VEGF, vascular endothelial growth factor; ANG, angiogenin; CTSS, cathepsin S; ATGL, adipose triglyceride lipase; CGI-58, comparative gene indentification 58; G0S2, G0/G1 switch gene 2; HSL, hormone-sensitive lipase. ^*^VAL *vs.* PLB treatment assessed by repeated-measures ANOVA. Values are medians (interquartile range).

### ATBF and AT Gene Expression of Capillarization and Hypoxia Markers

**Figure 3:**
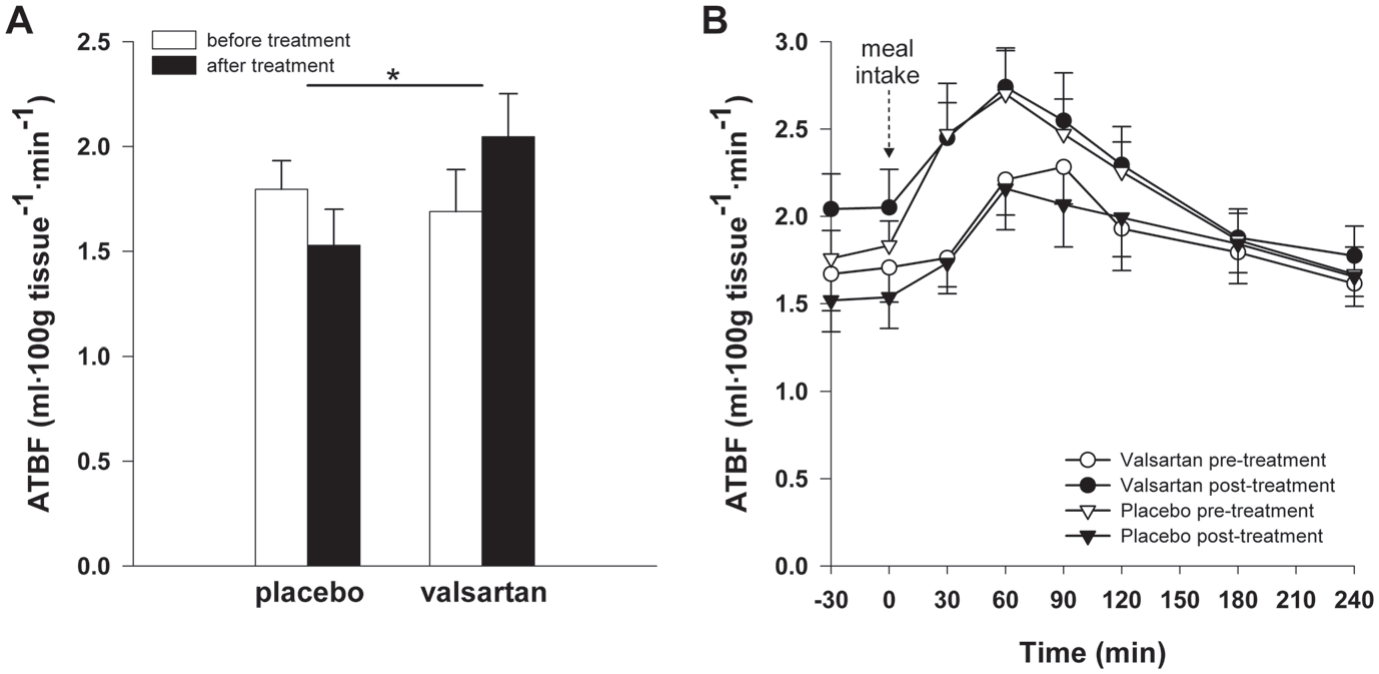
Fasting and postprandial ATBF VAL treatment (*n* = 16) significantly increased both (**A**) fasting ATBF and (**B**) postprandial ATBF (*P* = 0.049) compared with PLB (*n* = 14). A high-fat mixed-meal (containing 2.6 MJ, consisting of 61E% fat (35.5E% saturated fatty acids (FAs), 18.8E% monounsaturated FAs and 1.7E% polyunsaturated FAs), 33E% carbohydrate and 6E% protein) was ingested at t0 min. Values are means±SEM. *P<0.05 VAL vs. PLB. ATBF, adipose tissue blood flow.

Both fasting ATBF (VAL: 1.7±0.2 *vs.* PLB: 1.8±0.1 ml·100g tissue^−1^·min^−1^, *P* = 0.675) and the postprandial enhancement of ATBF (iAUC_ATBF_/min) after consumption of the high-fat mixed-meal (VAL: 0.21±0.11 *vs.* PLB: 0.37±0.12 ml·100g tissue^−1^ min^−1^, *P* = 0.349) were comparable before the start of treatment. VAL increased both fasting (*P* = 0.043) and postprandial (*P* = 0.049) ATBF compared with PLB (Figure 3A and B, respectively). Adjustment for non-significant baseline differences in ATBF between groups did not alter the results.

AT gene expression of vascular endothelial growth factor (VEGF), the master regulator of vasculogenesis, angiogenesis and remodeling of blood vessels [23], was decreased after VAL treatment compared with PLB (*P* = 0.051) (Table 2). In accordance, VAL reduced AT gene expression of the angiogenesis and capillarization markers CD34 (*P* = 0.037) and angiogenin (ANG) (*P* = 0.028) (Table 2). VAL treatment did not affect mRNA expression of the hypoxia marker GLUT-1 (P = 0.741).

### AT Chemoattraction, Macrophage Infiltration and Inflammatory Markers

Adipocyte size was positively associated with AT gene expression of chemoattraction, macrophage infiltration and inflammatory markers (data not shown). VAL treatment decreased AT gene expression of the macrophage infiltration markers CD68 (*P* = 0.014), CD163 (*P* = 0.023) and CD206 (*P* = 0.004) (Table 2). Furthermore, VAL decreased cathepsin S (CTSS) (*P* = 0.014) AT mRNA expression (Table 2), which may reflect an improved inflammatory state of AT [24]. AT gene expression of monocyte-chemoattractant protein (MCP)-1 (*P* = 0.202), IL-6 (*P* = 0.426), TNF-α (*P* = 0.464) plasminogen activator inhibitor (PAI)-1 (*P* = 0.476) and adiponectin (*P* = 0.393) was not altered.

The change in adipocyte size after VAL treatment was associated with alterations in AT gene expression of CD68 (*r* = 0.639, *P* = 0.010), CD11b (*r* = 0.539, *P* = 0.033), CD163 (*r* = 0.514, *P* = 0.050), CD206 (*r* = 0.504, *P* = 0.056), CTSS (*r* = 0.648, *P* = 0.017) and TNF-α (*r* = 0.468, *P* = 0.091).

### Systemic Inflammation

**Figure 4:**
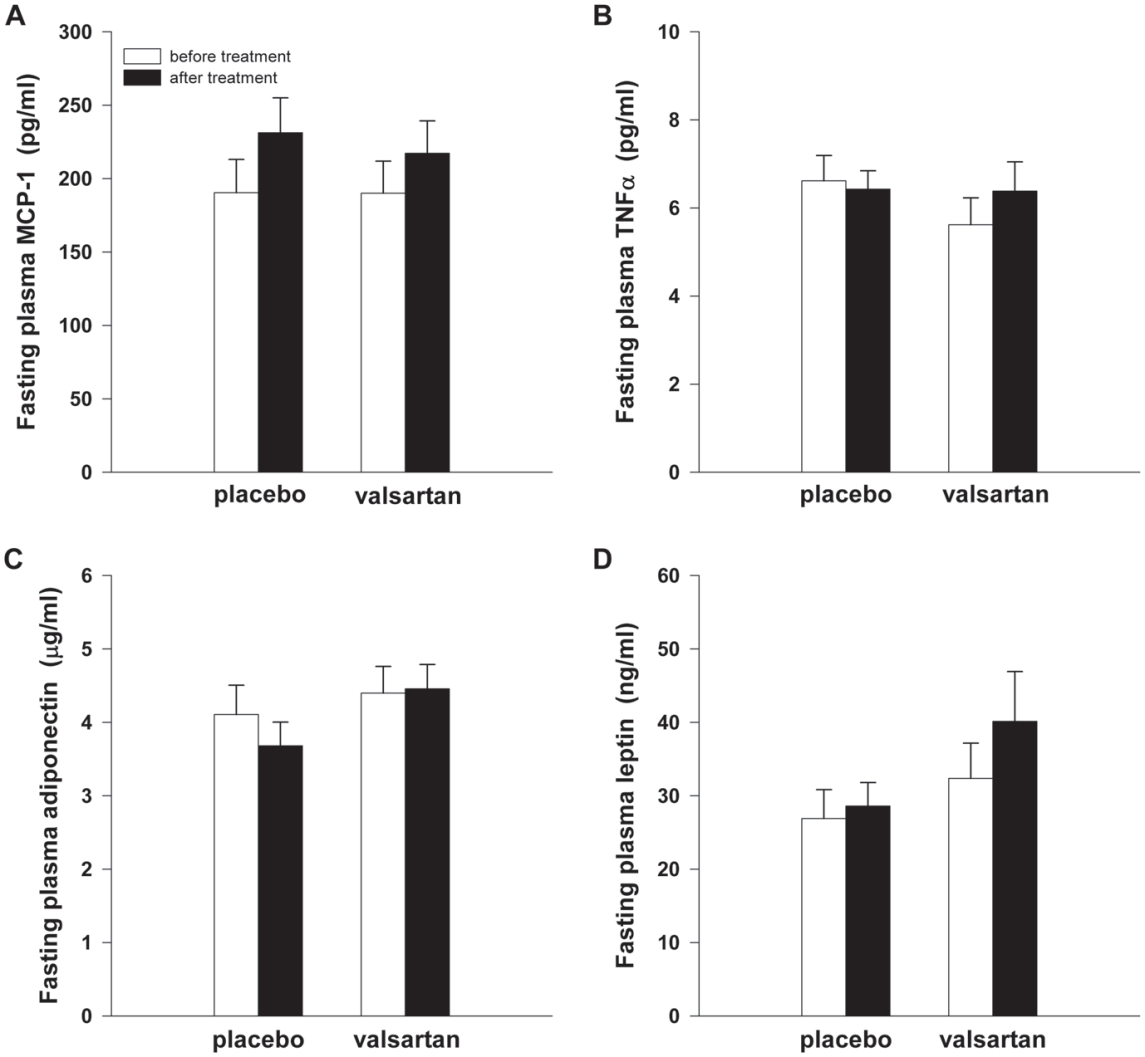
Circulating inflammatory markers VAL treatment (*n* = 17) did not significantly affect plasma concentrations of (**A**) MCP-1, (**B**) TNF-α, (**C**) adiponectin and (**D**) leptin compared with PLB (*n* = 19). MCP-1, monocyte chemoattractant protein-1; TNF-α, tumour necrosis factor-α.

At baseline, fasting plasma MCP-1 (*P* = 0.994), TNF-α (*P* = 0.243), adiponectin (*P* = 0.595) and leptin (*P* = 0.380) concentrations were comparable between groups (Figure 4A-D). VAL treatment did not significantly alter plasma concentrations of MCP-1 (*P* = 0.497), TNF-α (*P* = 0.106), adiponectin (*P* = 0.312) and leptin (*P* = 0.117) compared with PLB (Figure 4A-D).

### AT Expression of Lipolytic Enzymes and Co-factors involved in Lipolysis

VAL treatment did not significantly alter AT gene expression of the lipolytic enzyme adipose triglyceride lipase (ATGL) (*P* = 0.083), its activator protein comparative gene indentification 58 (CGI-58) (*P* = 0.090), G0/G1 switch gene 2 (G0S2) (*P* = 0.092) - which may attenuate ATGL action [25] – and hormone-sensitive lipase (HSL) (*P* = 0.710) compared with PLB (Table 2).

In line, AT protein expression of ATGL (*P* = 0.335), CGI-58 (*P* = 0.947), G0S2 (*P* = 0.299) and HSL (*P* = 0.821) was not altered after VAL treatment compared with PLB.

### Insulin Sensitivity

**Figure 5:**
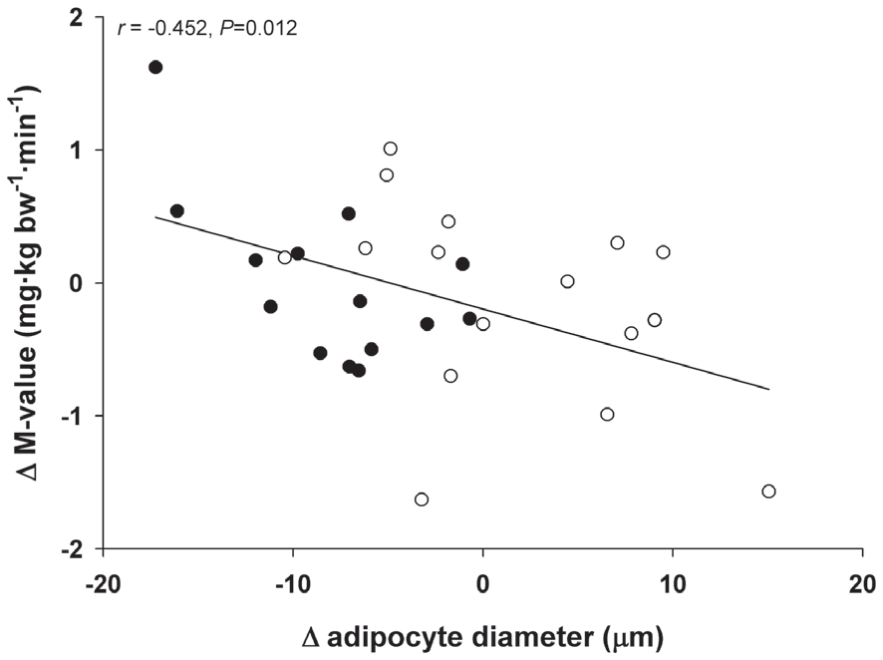
Correlation between the change in adipocyte diameter and insulin sensitivity after 26-wks VAL or PLB treatment (*n* = 30) The decrease in adipocyte size was significantly associated with alterations in insulin sensitivity after VAL (n = 14, closed circles) and PLB (n = 16, open circles) treatment (*r* = −0.452, *P* = 0.012).

The present study was conducted within the framework of the PRESERVE study, in which we have recently demonstrated that 26-wks VAL treatment significantly increased insulin sensitivity [15]. Although the change in insulin sensitivity after VAL treatment was of the same order of magnitude (∼10%) in the subset of subjects that was studied here (*n* = 38) compared with the total study population (*n* = 79), the VAL-induced increase in insulin sensitivity did not reach statistical significance in the present analysis (*P* = 0.248; Table 1). Importantly, the present study was not powered to detect a VAL-induced change in insulin sensitivity. Interestingly, however, treatment-induced alterations in adipocyte size were significantly correlated with changes in insulin sensitivity (*r* = −0.452, *P* = 0.012) (Figure 5).

## Discussion

Recent large clinical trials have demonstrated that RAS blockade reduces the incidence of type 2 diabetes [5], [6]. We have recently shown that this may be explained by improved insulin sensitivity and beta-cell function in subjects with IGM [15]. The present study demonstrated that 26-wks VAL treatment markedly reduced abdominal subcutaneous adipocyte size and AT macrophage infiltration markers, and increased ATBF in IGM subjects. The VAL-induced decrease in adipocyte size was associated with reduced expression of macrophage infiltration markers in AT.

We have previously shown that local administration of Ang II, the active component of the RAS, in abdominal subcutaneous AT decreased [19], [26], whereas local infusion of the ARB losartan increased ATBF in humans [19]. We therefore hypothesized that long-term oral ARB treatment may increase ATBF. Indeed, the present data showed that 26-wks VAL treatment increased both fasting and postprandial ATBF compared with PLB. As anticipated, the magnitude of the ATBF increase in the present study (∼35%) was lower than that observed during local ARB administration into AT (∼55%) [19]. Previous studies from our laboratory and others have demonstrated that both fasting ATBF and the postprandial increase in ATBF are decreased in obese, insulin resistant subjects [27]–[29], which was closely associated with insulin resistance [27], [29]. In fact, it has previously been shown that increased ATBF improved AT lipid handling via an enhanced triacylglycerol clearance [30] and decreased reesterification of non-esterified fatty acids [31], thereby preventing an excessive flux of lipids toward non-adipose tissues (ectopic fat storage) and, as a consequence, insulin resistance [8]. Therefore, the increased ATBF after VAL treatment may have contributed to increased insulin sensitivity, although the lack of a significant association between the VAL-induced increase in ATBF and alteration in insulin sensitivity in the present study may argue against this. AT gene expression of angiogenesis/capillarization markers was significantly reduced after VAL treatment, which may reflect a lowered angiogenic response secondary to the increase in ATBF. Recent data from our group [27] and others [32] suggest that oxygen tension in human AT ranges from 3.2–11.3%, which might not be low enough to activate the HIF-1α pathway [33]. This, together with the present findings of unchanged GLUT1 mRNA expression in AT after VAL treatment, suggests that it is highly unlikely that modulation of the HIF-1α pathway has contributed to the VAL-induced improvement of AT function.

Interestingly, we found that 26-wks VAL treatment markedly reduced mean adipocyte diameter and increased the proportion of small adipocytes. Abdominal subcutaneous adipocyte hypertrophy appears to be an independent marker of insulin resistance [34]. The reason for this may be that hypertrophic adipocytes have a reduced lipid buffering capacity and show disturbances in adipokine expression, with a shift toward a more proinflammatory phenotype [8], [27]. Thus, we speculate that VAL treatment may have evoked beneficial changes in the adipocyte secretory profile associated with smaller adipocytes, which in turn may have contributed to favorable alterations in metabolism. In addition, we found a decreased gene expression of macrophage infiltration markers in AT after VAL treatment. Interestingly, the VAL-induced change in adipocyte size was strongly associated with alterations in insulin sensitivity and AT gene expression of both M1 (CD11b) and M2 (CD163 and CD206) macrophage infiltration markers, which underlines the importance of adipocyte size reduction in adipose tissue function. These findings are in agreement with previous studies in rodents, showing that ARB treatment reduced adipocyte size and AT inflammation, and improved glucose homeostasis [9]–[14]. Of note, the change in insulin sensitivity was not a primary outcome parameter in the present study, which may explain why the VAL-induced increase in insulin sensitivity did not reach statistical significance, in contrast with our recent findings in a larger group of subjects with IGM [15].

The reduction in AT gene expression of macrophage infiltration markers did not translate into significant alterations in circulating MCP-1, TNFα, leptin and adiponectin concentrations in the present study. Importantly, however, we cannot exclude that the reduction in macrophage infiltration markers decreased the expression and/or secretion of other adipokines that have been linked to insulin resistance. In accordance with the present findings, it has been shown that ARB treatment had no effects on circulating leptin, TNF-α and adiponectin, despite increased insulin sensitivity [35]. In contrast, other studies have demonstrated alterations in circulating adipokines after ARB treatment [36], [37]. These apparently conflicting findings may be explained by differences in study population, since in several of these studies patients with essential hypertension and/or increased systemic inflammation participated.

Based on cell experiments and studies in rodents [11], [13], [14], [38], we hypothesized that stimulation of adipocyte differentiation may underlie the VAL-induced decrease in adipocyte size. We found that AT gene expression of the adipocyte differentiation marker PPARγ was significantly reduced, rather than increased, after VAL treatment. Although this finding was surprising, it may well be that adipocyte size was already reduced several weeks before the end of treatment, which may in turn have down-regulated PPARγ expression, since the decrease in adipocyte size was strongly associated with reduced PPARγ expression after VAL treatment. Unfortunately, we did not collect AT biopsies at multiple time-points during the treatment period to confirm this.

Important processes in the regulation of adipocyte size, in addition to adipocyte differentiation, include the storage and release of fatty acids. In accordance with previous findings showing increased lipid synthesis and storage in 3T3-L1 and human adipocytes during Ang II stimulation [39], we found that VAL treatment reduced AT gene expression of aP2 and C/EBPα, which are key regulatory factors in adipogenesis and lipogenesis [22]. These data may suggest that decreased AT lipogenesis underlies the VAL-induced decrease in adipocyte size. Furthermore, we assessed whether VAL affects lipolysis, since previous findings from our group indicate that Ang II exerted modest inhibitory effects on AT lipolysis in humans [26]. However, both AT gene and protein expression of several lipolytic enzymes were not altered by VAL. These data indicate that it is unlikely that increased AT lipolysis has contributed to a significant extent to the VAL-induced decrease in adipocyte size in the present study. However, we cannot fully exclude that lipase activity was stimulated during the early phase of treatment.

In conclusion, we demonstrated that 26-wks VAL treatment markedly reduced abdominal subcutaneous adipocyte size and AT macrophage infiltration markers, and increased ATBF in IGM subjects. The VAL-induced decrease in adipocyte size was associated with reduced expression of macrophage infiltration markers in AT. In other words, our data indicate that VAL treatment was able to prevent further deterioration of AT function in subjects with IGM. Thus, improved AT function may underlie the increased insulin sensitivity [15] and reduced incidence of type 2 diabetes after long-term ARB treatment in subjects at high-risk of developing this disease. Furthermore, our findings imply that other interventions targeting the RAS may improve AT function, thereby lowering the risk for cardiovascular disease and type 2 diabetes.

## Supporting Information

Checklist S1 CONSORT Checklist.
(PDF)

Protocol S1 Trial Protocol.
(PDF)
