# Supplementary material for: Valsartan Improves Adipose Tissue Function in Humans with Impaired Glucose Metabolism: A Randomized Placebo-Controlled Double-Blind Trial
Source: PLoS One. 2012 Jun 29;7(6):e39930. doi: 10.1371/journal.pone.0039930 (PMC3386933; doi:10.1371/journal.pone.0039930)
Supplement: Protocol S1 — Trial Protocol. (PDF) [file pone.0039930.s002.pdf]

Version: 16 November 2006

PRESERVE TRIAL
Pancreatic β-cell dysfunction REStorEd by Rosiglitazone and Valsartan Effects

A 52-week randomized controlled factorial study in subjects with IFG and/or IGT

EudraCt nummer: 2006-003483-59

Investigators:
VU University Medical Center
M. Diamant, Diabetes Center / Endocrinology, VUMC, Amsterdam
R.J. Heine, Diabetes Center / Endocrinology, VUMC, Amsterdam
N.J. v.d. Zijl, Diabetes Center / Endocrinology, VUMC, Amsterdam
G. Nijpels, EMGO / University General Practice, VUMC, Amsterdam
J.M. Dekker, EMGO, VUMC, Amsterdam
P.J. Kostense, Clinical Epidemiology and Biostatistics/EMGO, VUMC, Amsterdam
Y.M. Smulders, Internal Medicine, VUMC, Amsterdam
E.H. Serné, Internal Medicine, VUMC, Amsterdam

Maastricht University
E.E. Blaak, Dept. of Human Biology (NUTRIM), Maastricht University
M. van Baak, Dept. of Human Biology (NUTRIM), Maastricht University
G.H. Goossens, Dept. of Human Biology (NUTRIM), Maastricht University

Correspondence:
Dr. M. Diamant, Associate Professor of Medicine
Prof. dr. R.J. Heine, Professor of Diabetology, Director of Diabetes Center VUMC
Diabetes Center / Endocrinology
VU University Medical Center
De Boelelaan 1117
PO BOX 7057
1007 MB Amsterdam
phone: +31-20-4440533
fax: +31-20-4440502
e-mail: m.diamant@vumc.nl

STUDY SUMMARY

Worldwide, type 2 diabetes mellitus is a major and growing health problem reaching epidemic proportions. DM2 is characterized by insulin resistance and progressive β-cell failure, with the latter accounting for the progressive course of the disease. Current therapies fail to prevent the progressive nature of DM2, since no treatment exists that can effectively prevent or slow the decline of β-cell function.

The PRESERVE (Pancreatic β-cell dysfunction REStorEd by Rosiglitazone and Valsartan Effects) Trial is designed to determine whether treatment with rosiglitazone and/or valsartan will favorably affect β-cell function in subjects with impaired fasting glucose (IFG; blood glucose ≥ 5.6 and < 7 mmol/l) and/or impaired glucose tolerance (IGT; 2-h post-load glucose 7.8-11.1 mmol/l). Since the two interventions have different mechanisms of action, it may be expected that the effects will be independent and potentially complementary (additive).

Rosiglitazone improves insulin sensitivity but may also have β-cell protective effects, by lowering triglyceride accumulation in pancreatic islets, decrease inflammation and collagen deposition and anti-apoptotic actions. Valsartan may prevent deterioration of β-cell function by interference with the renin-angiotensin system, both systemically and locally within the pancreatic islets. In particular, the latter action may reduce islet inflammation and scarring and increase pancreatic local blood flow. Other beneficial effects of generalized RAS blockade include an increase in disposal of glucose, a decrease in sympathetic nervous tone and the prevention of ectopic triglyceride accumulation by increasing adipogenic differentiation.

The PRESERVE study is a multi-center, randomized double-blind double-dummy trial using a balanced 2 x 2 factorial design in approximately 144 subjects with IFG (with and without a family history of DM2) or IGT. After randomization, subjects will be treated for 52 weeks, then study medication will be discontinued. At 3 months after discontinuation of the study medication, β-cell function will be re-evaluated to assess durability / disease modifying effects of the study drugs. Beta-cell function will be measured using modified euglycemic-hyperglycemic clamps prior to randomization, at 52 weeks and at 64 weeks.
The primary study endpoint is the treatment effect on β-cell function as measured by changes in glucose induced first phase insulin secretion corrected for changes in insulin sensitivity and / or the (first phase) arginine-stimulated insulin secretion during a hyperglycemic clamp.
Secondary study endpoints include changes in fasting plasma glucose, the second phase insulin secretion in response to hyperglycemia during the clamp, conversion of NGT to IGT or diabetes, clamp-measured whole body insulin sensitivity, anthropometric and metabolic / lipid variables, as well as markers of vascular damage, inflammation and endothelial function. Those patients developing diabetes during the study (i.e. a diagnosis of diabetes will be made if 2 consecutive fasting plasma glucose (FPG) levels exceed the diagnostic thresholds of ≥7.0 mmol/l (126 mg/dl) or a 2 hr plasma post-load glucose ≥11.1 mmol/l (200 mg/dl)) with FPG ≥ 10 mmol/l measured at 2 occasions, will be discontinued from the study after an end-point measurement.
Assuming an annual 5% deterioration of β-cell function in the dummy group and a 25% improvement in the treated groups (with SD=50%, power=0.8 and significance level=0.025, using the Hochberg procedure for multiple testing), we expect that a total of about 120 evaluable subjects will be sufficient. This means that 36 subjects per treatment group need to be included taking into account a drop-out rate of 20%.

TABLE OF CONTENTS

STUDY SUMMARY……………………………………………………………..………..…..…2
TABLE OF CONTENTS……………………………………………………………………..3
BACKGROUND AND RATIONALE………………………………………………...….4
PPARγ and the β-cell……………………………………………………………………4
The renin-angiotensin system……………………………………………………..4
Thiazolidinediones……………………………………………………………………4
Beta-cell function measurements in humans……………………………………..5
The metabolic syndrome or pre-diabetes…………………………………………5
Summary…………………………………………………………………………………6
HYPOTHESES……………………………………………………………………………..6
DESCRIPTION OF THE PRESERVE TRIAL……………………………………………..7
Study Objectives…………………………………………………………………………7
Description of Endpoints..……………………………………………………………7
Study Design……………………………………………………………………………..8
Study Population…..…………………………………………………………………..9
Study Medication………………………………………………………………………..9
Study Methods…………………………………………………………………….…10
Screening and pre-randomization phase…………………………………………..10
Randomization…………………………………………………………………………11
Follow-up……………………………………………….………………………11
Concomitant Medication and Concomitant Illness……………………………….11
Storage and Provision of Study Medications…………………….……..……………12
Emergency Unblinding of Treatment Assignment………………………………..12
Safety…………………………………………………………………………………12
Study Drug Discontinuation / Withdrawal from the Study………………………………13
Study Completion and Post-Study Treatment…..…………………………………13
Laboratory Procedures/ Blood Volume..……………………………………………..13
STATISTICAL CONSIDERATIONS…………………………………………….…….14
General Considerations……………………………………………………………..14
Analysis population…………………………………………………………………15
Study Endpoints / Primary Study Endpoint…………………………………………15
Justification of Sample Size…………………………………………………………14
Statistical Analysis……………………………………………………………………..15
SUBSTUDIES……………………………………………………………………………..16
ETHICS & LEGAL REQUIREMENTS……………………………………………………...16
REFERENCES…………………………………………………………………………16
Appendix 1: Study Plan Sheet……………………………………………………………19
Appendix 2: Safety Considerations and Reporting……………………………………..20
Appendix 3: Euglycemic-Hyperglycemic Clamp………………………………………..22
Appendix 4: Oral glucose tolerance test……………………………………………………24
Appendix 5: Body fat distribution/liver fat content assessed by MRI/^{1}H-MRS…………………25
Appendix 6: Fatty acid partitioning and body composition…………………………………... 27
Appendix 7: Carotid IMT/DC Assessments by Ultrasound…………………………………..31
Appendix 8: Microvascular function assessments…..…………………………………..…32

BACKGROUND

Type 2 diabetes mellitus (DM2) is characterized by insulin resistance and progressive pancreatic β-cell dysfunction.[1,2] The cornerstone of current oral blood-glucose lowering therapy consists of metformin, which primarily lowers hepatic glucose production, and the sulphonylureas that act by stimulating pancreatic β-cells to secrete insulin.[3] Therapeutic strategies targeting either insulin resistance and/or β-cell dysfunction, the two key mechanisms underlying the development of DM2, are currently being explored. However, at present there are no pharmacological therapies available that effectively prevent or slow the decline of β-cell function in pre-diabetic conditions, such as impaired fasting glucose (IFG) and impaired glucose tolerance (IGT) and/or the metabolic syndrome, or in subjects with overt DM2.

PPAR and the β-cell. PPARγ is expressed in the β cells of rodents and humans,[4,5] and treatment with PPARγ agonists leads to improvements in islet architecture, insulin content, and glucose-stimulated insulin secretion.[6,7] Although these effects could be explained by the consequences of improved peripheral insulin sensitivity, direct effects of these drugs on pancreatic islets have been shown in vitro. The mechanisms underlying these effects, however, remain unclear. Recent evidence indicates that PPARγ agonists directly activate genes of the glucose-sensing apparatus in pancreatic β-cells.[7] In addition, troglitazone restored β-cell function in Zucker diabetic fatty (ZDF) rats by lowering triglyceride accumulation in pancreatic islets.[8] Rosiglitazone treatment prevented β-cell death by anti-apoptotic actions in the prediabetic OLETF rat.[9] Another potentially relevant mechanism by which interference with the PPARγ may contribute to the preservation of β-cell function is the PPARγ-mediated regulation of inflammation and collagen accumulation.[10] However, the controversy continues as recent findings in mice with targeted elimination of PPARγ in β-cells showed that the presence of PPARγ in the pancreatic β-cell was not necessary for glucose homeostasis.[11]

The renin-angiotensin-system. The renin angiotensin system (RAS) is involved in the pathogenesis of obesity and insulin resistance, as well as the associated abnormalities including hypertension and endothelial dysfunction.[12-14] Insulin resistance has been implicated in the link between diabetes and hypertension. Angiotensin II (ATII) inhibits insulin signaling,[15] thus playing a role in insulin-regulated processes, in particular glucose homeostasis.[16] However, ATII may also influence glucose metabolism in an insulin-independent manner.[17,18] ATII plays an important role in the pathogenesis of atherosclerosis and cardiovascular disease (CVD).[13,14] Most of the vascular and metabolic effects are mediated by the ATII type 1 receptor (AR-I). ATII causes vascular smooth muscle cell (VSMC) hypertrophy, extracellular matrix production and the expression of various growth factors. In addition, in most tissues, chronic exposure to ATII increases oxidative stress, activates fibrogenesis and promotes apoptosis. All of these processes have also been implicated in the progressive loss of β-cell function in type 2 diabetes.[19] Indeed, the expression of the AR-I was increased more than threefold in the islets of ZDF rats, suggesting that chronic hyperglycemia and hyperlipidemia can activate the local RAS.[20] Chronic blockade of the RAS using the ACE-inhibitor perindopril or the angiotensin II-receptor blocker (ARB) irbesartan attenuated islet fibrosis, oxidative stress and apoptosis in these rats. In humans, several large trials have indicated that blockade of the RAS protects against the development of diabetes in hypertensive subjects who are at high-risk to develop type 2 diabetes.[21-26] In the recently published VALUE-study, the ARB valsartan, as compared with amlodipine, reduced the incidence of DM2 with 23% in subjects with hypertension.[27] Although, in general, these beneficial effects have been primarily attributed to drug-related improvements of peripheral insulin sensitivity, additional action may be expected at the level of the pancreatic β-cell. Clearly, studies are needed that address the mechanisms underlying the observed effects of these agents in high-risk populations.

Thiazolidinediones. About fifteen years ago, a novel class of agents, the thiazolidinediones (TZD), has been introduced.[28] TZD are synthetic ligands that bind to the nuclear peroxisome proliferator-activated receptor γ (PPARγ) receptor and exert their action by activating transcription of genes that, among others, regulate adipocyte differentiation and adipogenesis as well as glucose and lipid metabolism and inflammation.[10,28-30] In subjects with DM2, TZD (previously troglitazone, and

currently rosiglitazone and pioglitazone) given as monotherapy or in combination with sulphonylureas, metformin or insulin, improve glycemic control, by lowering fasting and postprandial blood glucose levels, and ameliorate insulin sensitivity in placebo-controlled trials.[28] Also, these agents exert beneficial effects on various CVD risk factors associated with insulin resistance, such as dyslipidemia, hypertension, body fat distribution, vascular function, microalbuminuria and inflammation.[28,31] Recent evidence indicates that TZD may also favorably affect several structural and functional aspects of pancreatic islets.[6-9,32-34]
In preclinical studies, TZD were shown to inhibit migration and proliferation of VSMC, NF-kB pathways that regulate expression of adhesion molecules, and PAI-I and endothelin-I expression and regulation.
Recent evidence indicates an important interaction between PPARγ-mediated signaling and the RAS. ATII infusion in rats resulted in hypertension, inflammation and oxidative stress all of which could be prevented by simultaneous treatment with TZD.[35] Activation of PPARγ downregulated the AR-I in VSMC. In view of these interactions, and the fact that ATII inhibits insulin signaling, it is feasible that the combined use of ARB and PPARγ agonists may have additive beneficial effects on β-cell function. Furthermore, these compounds may exert differential favorable effects on insulin sensitivity, (micro)vascular function and inflammation.

Beta-cell function measurements in humans. The nature and magnitude of TZD-induced effects on β-cell function have so far not been detailed in humans due to the complex interaction between insulin sensitivity and β-cell secretory function, but also, and perhaps most importantly, due to the lack of validated methods to reliably measure the different aspects of β-cell function.[36-40] A 12-week treatment with troglitazone resulted in improvement of the β-cell response to glucose in subjects with impaired glucose tolerance, as evaluated by graded and oscillatory glucose infusions and frequently sampled intravenous glucose tolerance test (IVGTT).[33] Moreover, 30 months of troglitazone therapy ameliorated β-cell function, as measured by IVGTT and oral glucose tolerance test, and ultimately delayed the onset of DM2 in a high-risk Hispanic women.[34] Still, the methods used in these studies may not be suitable to measure all relevant aspects of β-cell function and as such, may fail to reveal the full potential of TZD in modifying β-cell function in prediabetic subjects and patients with DM2. The hyperglycemic clamp is regarded as the gold standard to assess glucose-stimulated insulin secretion of the β-cell.[36] Other methods available for measuring insulin secretion include the IVGTT,[37] the continuous infusion of glucose with model assessment (CIGMA)[38] and intravenous glucagon test.[39] Addition of non-glucose secretagogues, including the amino acid arginine and the incretin hormone glucagons-like peptide (GLP)-1, at various levels of glycemia, provides further information on the glucose-sensing as well as insulin-producing and secretory capacities of the β-cell.[41,42] The (modified) clamp methodology at present seems to be the most reliable and reproducible method for β-cell function testing, although recently, β-cell function parameters were modeled from variables obtained after exposure to a standardized test-meal.[43] The advantages of this approach include the use of a physiological stimulus, the relative short duration of the test, no infusions and less blood collections. However, this tests requires further validation i.e. comparison with the gold standard clamp method, in different populations, such as in obese subjects, people with impaired glucose tolerance, subjects with impaired fasting glucose and the metabolic syndrome and patients with newly-diagnosed DM2.[44-46] Recently, a new protocol to assess β-cell function parameters from an OGTT has been validated.[46a]

The metabolic syndrome or pre-diabetes. In order to identify subjects at high risk to develop type 2 diabetes and CVD, the NCEP/ATPIII has suggested a cluster of abnormalities related to insulin resistance to define the so-called metabolic syndrome, also termed pre-diabetes.[44] These abnormalities include increased waist circumference, systolic blood pressure, plasma triglycerides, low HDL and abnormal glucose tolerance. The presence of 3 or more of these criteria are diagnostic for the metabolic syndrome. In some populations, the NCEP/ATPIII defined metabolic syndrome is an independent predictor of type 2 diabetes,[45] whereas it is insensitive in predicting diabetes in others.[46] Conversely, a high proportion of DM2 subjects fulfill the criteria of metabolic syndrome,[47]
and the presence of the metabolic syndrome was shown to increase the risk of diabetes-related

complications.[48]
The various metabolic abnormalities in these subjects, including central obesity, insulin resistance, hypertriglyceridemia and low-grade inflammation are known to adversely influence β-cell function. TZD are known to improve or normalize these disturbances.[28] Indeed, it was shown that TZD treatment improved β-cell function and ultimately delayed the onset of diabetes in high-risk populations.[34,49] Subjects with the metabolic syndrome are at high risk of CVD.[50,51] In addition to the presence of fore-mentioned (established) CVD risk factors, these subjects have a high prevalence of endothelial dysfunction, hypercoagulability and low-grade inflammation.[52] Treatment with TZD not only improves metabolic abnormalities but was also shown to reduce circulating markers of inflammation in subjects with DM2.[53,54] Furthermore, based on above-mentioned pathophysiological considerations and preliminary data from trials, pharmacological blockade of the RAS using ACE-inhibitors or ARB, may also be beneficial in subjects with early signs of β-cell function and the metabolic syndrome.
However, as yet, the observed PPARγ-mediated vascular effects in humans are not consistent: in some populations TZD improved endothelial function, whereas this was not found in other patient groups.[55,56] In addition, several large-scale prospective trials using PPARγ agonists and/or inhibitors of the RAS, such as the DREAM and NAVIGATOR trials, are ongoing to show the beneficial effects of these agents in people at high-risk of DM2 and CVD.[57] As mentioned before, the next important step is to study the mechanisms underlying the effects of these drugs in relevant populations.

To summarize, treatment strategies in people at high risk of developing DM2 as well as in those with newly-diagnosed DM2, should not only target insulin resistance and the associated abnormalities but also employ therapies that may prevent further β-cell function loss and reduce CVD risk. PPARγ agonists and ARB seem additional beneficial options for the treatment of subjects with early signs of β-cell dysfunction (i.e. those with IFG, with or without a family history of DM2, IGT with/without the metabolic syndrome). It may be expected that, by using a modified clamp method, abnormalities of the different aspects of β-cell function may be detailed as well as the modulation of these parameters by the proposed therapeutic interventions. The possible mechanisms by which these agents modulate β-cell function in humans may be further investigated by measurement of additional variables, including glucose and lipid metabolism, peripheral insulin sensitivity, body composition as well as markers of inflammation and vascular function. This approach will enable us to further explore the working spectrum of these important classes of agents.

HYPOTHESES

I. In addition to ameliorating insulin resistance, PPARγ agonist therapy may delay the progression of β-cell dysfunction in subjects with IFG and the metabolic syndrome by lowering triglyceride-accumulation in the β-cell, thus ameliorating β-cell lipotoxicity, and by mechanisms such as PPARγ-mediated reduction of inflammation, collagen accumulation and anti-apoptotis.

II. The RAS is involved in the pathophysiology of insulin resistance but is also implicated in progressive decline of β-cell function, which is a hall-mark of DM2 and which is fundamental for the transition from IFG/metabolic syndrome to overt DM2. Blocking the RAS by an ARB may therefore be expected to delay the progression of β-cell dysfunction in subjects with IFG and the metabolic syndrome.
The beneficial effects of blocking the AR-I may be effectuated by: a) an increase in disposal of glucose (by increasing transcapillary glucose transport); b) prevention of ectopic triglyceride deposition by increasing adipogenic differentiation; c) a decrease in sympathetic tone; d) an increase in pancreatic local blood flow; e) an increase in glucose-stimulated insulin release and f) a reduction of pro-inflammatory responses and scarring.

III. PPARγ agonists inhibit ATII-induced actions by down regulating tissue AR-I. Combined treatment with a PPARγ agonist and an ARB will have additional beneficial effects on β-cell function and as such not only prevent the decline of β-cell dysfunction but may even improve (several aspects of) β-cell function in subjects with IFG and/or IGT.

DESCRIPTION OF THE PRESERVE TRIAL

STUDY OBJECTIVES

PRIMARY
To compare β-cell function, as reflected by the first phase insulin secretion corrected for insulin sensitivity and/or the arginine-stimulated insulin secretion, both co-primary endpoints as measured during the eu-hyperglycemic clamp procedure, following 52 weeks of rosiglitazone, valsartan or rosiglitazone combined with valsartan in subjects with IFG (with and without a family history of DM2) and/or IGT.

SECONDARY*
To compare the effects of 52 weeks of rosiglitazone, valsartan or rosiglitazone combined with valsartan in subjects with IFG (with and without a family history of DM2) and/or IGT with respect to:

- Fasting plasma glucose
- Second phase insulin secretion in response to hyperglycemia during the hyperglycemic clamp test
- All the above-mentioned β-cell function parameters at 12 weeks after discontinuation of therapy to assess durability/disease modifying effects
- The conversion from normal glucose tolerance (NGT) to IGT or diabetes (as evaluated by an oral glucose tolerance test)
- HbA1c, fasting blood glucose and lipid/lipoprotein concentrations
- Insulin sensitivity assessed during the euglycemic clamp test
- Safety and tolerability, including assessments of hypoglycemic events, blood pressure, and urinary albumin excretion rate

EXPLORATORY VARIABLES

- Homeostasis model assessments of insulin sensitivity and β-cell function
- Circulating markers of inflammation and endothelial function
- Plasma levels of incretins, ghrelin and PYY

*** Secondary endpoints derived from the 3 substudies are described in Appendix 5-8**

DESCRIPTION OF ENDPOINTS
PRIMARY ENDPOINTS

Blood samples (ranging from 3-6 mL) will be drawn at specified time points during the eu-hyperglycemic clamp tests at Visit 2 (Week -2) and Visit 11 (Week 51), for measurement of insulin concentrations in order to calculate the first phase insulin secretion (i.e. incremental insulin AUC 0-10 min in response to glucose challenge) corrected for insulin sensitivity (M/I obtained during the last 30 min of the hyperinsulinemic euglycemic clamp) and/or the arginine-stimulated insulin secretion (i.e. i.e. incremental insulin AUC during the first 10 min in response to the arginine bolus), both co-primary endpoints measured during the clamp procedure. Samples will be stored at -80°C until analyzed. Refer to Appendix 4 for details regarding the clamp procedures.

SECONDARY ENDPOINTS

The change in co-primary endpoints at week 64 as compared to week 52 and baseline.

To assess durability and disease-modifying effects of both agents

Second Phase Insulin Release in Response to Hyperglycemia

The change from baseline to 1 year in the second phase insulin release during hyperglycemia. See Appendix 4 for details regarding the euglycemic/hyperglycemic clamp procedures.

Circulating Markers of Inflammation, Coagulation, and Endothelial Function

Blood samples (up to approximately 15 mL) will be drawn at Visit 3 (Week -1), Visit 11 (Week 51), and Visit 15 (Week 64) for the measurement of adipocytokines (leptin, adiponectin, resistin) and markers of inflammation (hsCRP, IL-6, TNFα, MCP-I, MMP) and endothelial function (Von Willebrand Factor, E-selectin), as well as spare samples for determinations of emerging markers relevant to the study objectives, e.g. such as those related to β-cell mass (TMEM27). Samples will be stored at -80°C until analyzed.

Fasting Plasma Glucose, HbA1c

Blood samples (2 mL) will be drawn for the assessment of fasting plasma glucose at Visit 1 (Screening), Visit 4 (Day 1), Visit 6 (Week 8), Visit 7 (Week 16), Visit 8 (Week 24), Visit 9 (Week 32), Visit 10 (Week 40), Visit 12 (Week 52), Visit 13 (Week 56/58), and Study Termination (Week 64) or early termination, if applicable. Blood for the analysis of HbA1c is obtained from the hematology blood draw tube.

Fasting Plasma Lipids

Fasting plasma lipids (total cholesterol, HDL cholesterol, LDL cholesterol and triglycerides) at will be measured from blood drawn at Visit 1 (Screening), Visit 4 (Day 1), Visit 6 (Week 8), Visit 7 (Week 16), Visit 8 (Week 24) and Visit 12 (Week 52). If an early termination visit occurs prior to Visit 12 (Week 52), samples will be collected for measurement of fasting plasma lipids. Blood for these analyses is obtained from the clinical chemistry blood draw tube.

Oral Glucose Tolerance Test; Conversion from NGT to IGT or Diabetes

A 75-g oral glucose tolerance test (OGTT) will be performed after a 12-h overnight fast at Visit 1 (Screening), Visit 3 (Week -1), Visit 12 (Week 52) and Study Termination (Week 64).

Insulin Sensitivity

Insulin sensitivity will be assessed with the euglycemic clamp part of the eu-hyperglycemic clamp procedure at Visit 2 (Week -2), Visit 11 (Week 51), and Visit 14 (Week 62/63). Insulin sensitivity will be expressed as the insulin sensitivity index (ISI), which is calculated by dividing the average glucose infusion rate (in mmol/[kg*min]) during the last 30 minutes of the euglycemic clamp portion of the euglycemic/hyperglycemic clamp test, by the average plasma insulin concentration (in pmol/L) during the same time interval. Refer to Appendix 4 for the related procedures.

Homeostasis Model Assessment

Treatment effects of rosiglitazone and valsartan or combination of the drugs on homeostasis model assessment (HOMA) will be examined as an exploratory variable. The pancreatic beta-cell function (%HOMA-B) and peripheral and hepatic insulin sensitivity (%HOMA-S) will be determined with a computerized HOMA model using fasting plasma glucose and insulin concentrations determined at Visit 2 (Week -2), Visit 11 (Week 51), and Visit 14 (Week 62/63).

STUDY DESIGN

A randomized controlled, balanced 2 x 2 factorial design, which allows to evaluate two factors simultaneously with fewer subjects than needed in 2 separate studies with parallel design, will be performed. After a 4-week run-in period during which patients will be instructed with regard to lifestyle modifications and during which the baseline measurements will be performed, subjects will

be randomized to one of the four groups defined by Rosiglitazone/Valsaratan-dummy, Valsartan/Rosiglitazone dummy, Rosiglitazone/Valsartan or double dummy/dummy, as outlined below:

Treatment group:	1	2	3	4
Intervention:	Rosiglitazone	R-dummy	Rosiglitazone	R-dummy
	V-dummy	Valsartan	Valsartan	V-dummy

R=rosiglitazone; V=valsartan

STUDY POPULATION

Subjects:

One-hundred and forty-four male and female subjects (aged 35-70 years) with impaired fasting glucose (IFG; plasma glucose ≥ 6.1 and < 7.0 mmol/l) and/or subjects with IFG (plasma glucose ≥ 5.6 and < 7.0 mmol/l) ***and*** a family history of DM2 (i.e. first and second degree (i.e. grandparents) relatives), and/or impaired glucose tolerance (IGT; 2-h plasma glucose during 75-g OGTT 7.8-11.1 mmol/l) are eligible. Patients will be recruited in 3 regions, i.e. region of Amsterdam, from the Hoorn Screening Study (see below), in the region of Maastricht – among others through advertisements.

The **exclusion criteria are as follows:**

Criteria	Definition of Criteria
Drug Use	a) current use of ACE-I, ARB and/or TZDs and inability to discontinue these medications<br>b) known hypersensitivity to any of the study drugs<br>c) prior use of blood glucose lowering medications except during pregnancy<br>d) use of systemic glucocorticoids or niacin
Cardiovascular Disease	a) ejection fraction known to be <40% or congestive heart failure, or existing clinical CV disease (previous MI or stroke; angina with either >50% stenosis in >=2 major coronary arteries, or ST depression of >=2mm, or a positive nuclear test, previous coronary angioplasty, stent or bypass; previous limb bypass or vessel angioplasty or angiographic evidence of >50% stenosis, or intermittent claudication with an ankle/arm pressure <=0.8)<br>b) uncontrolled hypertension requiring ACE I or ARB
Other Criteria	a) History of diabetes (except gestational DM) or on antidiabetic medication<br>b) Renal or Hepatic Disease<br>i) renal artery stenosis<br>ii) creatinine clearance <40 ml/min or serum creatinine ≥200 umol/l<br>iii) clinical proteinuria (≥1+ proteinuria on dipstick or ≥300 mg of albuminuria/day, in the absence of urine)<br>iv) measured alanine transferase (ALT) ≥2.5 times the upper limit of normal<br>v) active liver disease including jaundice, chronic hepatitis, previous liver transplant<br>c) Major illness with life expectancy < 5 years or that may interfere with participation<br>d) Use of another experimental drug<br>e) Pregnant or unwilling to use reliable contraception (fertile women will have a pregnancy test prior to randomization)<br>f) Major psychiatric disorder<br>g) Diseases and medications that affect glucose tolerance (e.g. pheochromocytoma, Cushing's syndrome, acromegaly, steroid-dependent asthma, protease inhibitors, antipsychotics)<br>h) Unwillingness to be randomized or sign informed consent<br>i) Known uncontrolled substance abuse<br>i) Inability to understand study information and/or communicate with clinic staff

STUDY MEDICATION

Study medication will be blinded and consists of rosiglitazone (RSG) 8 mg once daily (to be taken as 2 tabs of 4 mg RSG once daily), valsartan (VAL) 320 mg once daily (to be taken as 2 caps of 160 mg VAL once daily) and matching placebo tablets.

After randomization, a 2-week titration period will be observed: i.e. RSG will be started at a dose of 4 mg once daily and escalated to the full dose of 8 mg daily (to be taken as once daily 2 tabs of 4 mg) after 2 weeks; VAL will be started at a dose of 160 mg once daily and escalated to the full dose of 320 mg daily after 2 weeks. Patients will receive their study medication (2 bottles clearly marked to avoid mistakes) at their visits to the clinic and they will be instructed to take 1 tab once daily from each bottle during the titration period and 2 tabs once daily from each bottle during the rest of the study period. Study medication should be taken at bed-time, other medications, e.g. blood-pressure lowering therapy, should be taken in the morning (see below). The patient packs will be prepared by the hospital pharmacy according to a randomization scheme with block size of 12 (consisting of 3 x 4 different treatment regimens). Each study drug and respective placebo will be packaged into separate bottles containing the number of tablets (RSG or matching placebo) or capsules (VAL or matching placebo) to be taken each day, times the number of days between 2 visits (+ 7 extra daily doses of each drug/placebo). Four different packs will be prepared, corresponding with the 4 different treatment groups::

Pack A: One bottle containing tabs of RSG 4 mg verum plus one bottle containing caps of VAL placebo
Pack B: One bottle containing tabs of RSG placebo plus one bottle containing caps of VAL 160 mg verum
Pack C: One bottle containing tabs of RSG 4 mg verum plus one bottle containing caps of VAL 160 mg verum
Pack D: One bottle containing tabs of RSG placebo plus one bottle containing caps of VAL placebo

During the titration period, the participants will be instructed to take one tab/cap from each bottle, during the rest of the study period, they will be instructed to take 2 tabs/caps from each bottle. In case of down-titration, participants will be instructed to take only one tab/cap from each bottle.
Patient compliance will be monitored at the clinic visits by counting the unused number of tablets.

Visits to evaluate the primary and secondary objectives:
During one visit, the combined euglycemic-hyperglycemic clamp will be performed (duration: 6-7 h).
During a second visit, subjects will undergo a 75-g OGTT and additional blood collections.
In the case of sub-study 1 (quantification of intra-abdominal fat and liver fat), the OGTT will be preceded by an MRI/^{1}H-MRS (Amsterdam and Maastricht sites; see appendix 7). When sub-study 2 will be performed (Maastricht site; see appendix 6), muscle and fat tissue blood flow and lipolysis will be measured and fat and muscle biopsies will be obtained during a separate visit, before and after 52 weeks of therapy.

STUDY METHODS
An outline of study procedures is shown in the flow chart in Appendix 1:
I. Modified euglycemic – hyperglycemic clamps
II. Oral glucose tolerance test
III. MRI of abdominal adipose tissue and ^{1}H-MRS of the liver (sub-study 1)
IV. Fat partitioning and fat and biopsies (sub-study 2)
V. Carotid artery IMT and distensibility; microvascular function (sub-study 3)

SCREENING AND PRE-RANDOMIZATION OR PRE-STUDY PHASE
Participants will be recruited through advertisements and posters in waiting-rooms of general practices (e.g. subjects with obesity and hypertension). If necessary, all participating sites will attempt to recruit subjects from additional sources including first degree relatives of diabetic people in DM clinics, ads in newspapers, pharmacies, national diabetes associations, newsletters, and family practice clinics; community announcements; screening programs in workplaces and other defined populations; public presentations; and media stories.

The **standard screening visit** will consist of: questionnaires (about as used in the New Hoorn Screen Study, i.e. SQUASH (daily hassles and life events, habitual physical activity), smoking, alcohol

intake, employment, education, current medication, disease history, family history of disease, and self-reported birth weight), medical history, physical examination, anthropometrics, vital signs, ECG, 75-g OGTT, various baseline laboratory assessments, including a pharmacogenetics sample after obtaining separate informed consent from the subject, measurement of carotid IMT and distensibility by ultrasound (Amsterdam, Hoorn).

The procedures during screening and pre- randomization phase are listed in Appendix 1.
It is important that all subjects enter the study with a blood pressure < 140/90 mmHg, with or without antihypertensive drugs. Thus, if at screening uncontrolled hypertension or hypertension treated with other drugs than those listed below in the pre-specified algorithm is detected, patients will be asked to sign inform consent and enter a 12 week pre-study phase during which blood pressure will be treated according to the following algorithm:
Step 1: Amlodipine 5mg once daily (taken in the morning)
Step 2: Amlodipine 5mg once daily + hydrochlorthiazide (HCTZ) 12.5mg once daily
Step 3: Amlodipine 10mg once daily + HCTZ 12.5mg once daily (increase Amlodipine only at step 3 to avoid peripheral edema).
Step 4: Amlodipine 10mg once daily + HCTZ 12.5mg once daily + Carvedilol 25mg once daily.
If Amlodipine 10mg once daily cannot be tolerated **titrate back** to 5mg and add Carvedilol 25 mg once daily.

After three months of stable anti-hypertensive therapy and blood pressure < 140/90 mmHg, patients are eligible to participate in the study.

RANDOMIZATION
After review of their eligibility and final assessments (see Appendix 1), subjects will be randomized to either study drugs or matching placebo according to block randomization.

FOLLOW-UP VISITS
The procedures during the follow-up period are listed in Appendix 1. These include history taking, physical examination/anthropometric measurements, blood and urine collection for safety / outcome parameters.

CONCOMITANT MEDICATION AND CONCOMITANT ILLNESS
General
All participants and their primary care physicians will be provided with a list of all available ACE-Is, ARB and TZDs, and will be advised to avoid these drugs and inform the research staff if they are prescribed. As both thiazide diuretics and beta blockers can also cause glucose intolerance, participants and their physicians will be advised to withhold these agents unless they are specifically indicated (e.g. beta blockers post myocardial infarction). The 2 agents allowed from these respective categories (HCTZ at a maximum dose of 12.5 mg once daily and Carvedilol 25 mg once daily) are listed above in the pre-specified algorithm. Physicians will be advised to treat hypertension according to the above mentioned algorithm in order to reduce variability. If diabetes is diagnosed and requires pharmacologic therapy, antihyperglycemic agents other than a TZD will be prescribed.

For intercurrent events with proven indications for ACE-Is (e.g. congestive heart failure), or if a cardiovascular event occurs which increases the risk of a second event (e.g. myocardial infarction, documented peripheral vascular disease, angioplasty or stroke) the study drug should be stopped and the attending physician should consider the prescription of an ACE-I/ARB as necessary. If the attending physician chooses not to prescribe ACE-I/ARB, the subject may remain on study VAL/placebo.
TZD may exacerbate or cause fluid retention and edema (which may exacerbate heart failure in susceptible individuals such as those with poor LV function). Therefore, participants with symptomatic congestive heart failure (class 3 or 4), will be withdrawn from the TZD arm if they cannot be managed with diuretics or dose reductions of RSG study drug (but as the study participants are a relatively low risk group for any cardiovascular events, the incidence of heart failure is likely to

be less than 1 to 2% during the entire course of this trial). If either study drug is stopped for a reason not related to a serious adverse event (e.g. non-serious adverse event such as non-cardiac surgery, non-cardiac illness, hospitalization, azotemia, mild hyperkalemia) every attempt should be made to restart it at the same or lower dose, after adjustment of other medical therapy.

Detailed instruction on concomitant medication
The following drugs are not allowed during the study:

1. ***Anti-hypertensive drugs.*** No use of ACE-inhibitors or non-study ARB's is allowed. In order to become eligible, patients treated for hypertension with ACE-I or ARBs should switch to a calcium-channel blocker (amlodipine) and if necessary to additional drugs according to the above-mentioned algorithm, and the pre-study phase of 12 weeks should be observed. Patients already using diuretics or β-blockers for hypertension may be switched to the agents described in the algorithm, since several β-blockers and doses of HCTZ > 12.5 mg once daily may adversely affect the outcome parameters. Anti-hypertensive therapy should preferably be unchanged during the study, unless a clinical indication exists for adjustment. If anti-hypertensive therapy becomes indicated during the study (BP > 140/90 mm Hg, measured on 3 separate occasions), blood- pressure lowering therapy should be commenced according to the above-described algorithm.
2. ***Lipid lowering-drugs.*** No non-study PPAR γ or PPARα (fibrates) agonists are allowed. The use of statins is allowed.
3. ***Glucose-lowering agents.*** These include all sulfonylureas, biguanides, α-glucosidase inhibitors, non-study thiazolidinediones, repaglinide, nateglinide and insulin.
4. ***Chronic oral or parenteral corticosteroids treatment.*** (> 7 consecutive days of treatment).
5. ***Anti-cancer and/or cytostatic drugs.***

STORAGE AND PROVISION OF STUDY MEDICATIONS
Together with the trial pharmacist, the investigator will be responsible for ensuring that all study medication is stored in a dry location, below 25° C, protected from exposure to any environmental changes and in a locked facility; this will be a special place at the hospitals pharmacy. Only the investigator and trial pharmacist ('s assistant) will have access to the drug supplies. The pharmacist is responsible for randomization. After ending the 64 week study period, the code will be broken.

EMERGENCY UNBLINDING OF TREATMENT ASSIGNMENT
Emergency unblinding should only be done when necessary in order to treat the patient. Most often, study drug discontinuation and knowledge of the possible treatment assignments are sufficient to treat a study patient who presents with an emergency condition. Coded and sealed envelopes for each participant will be stored in a place where they are readily available to the study physician in case of a serious adverse event (SAE). In the case of a SAE or a medical emergency requiring knowledge of the study medication, the study physician will break the treatment code by opening the envelope. When the investigator breaks the code, he/she must note the date, time, and reason for removing it and retain this information with the case report form documentation. Reasons for breaking the code will be explained clearly and justified in writing to the Medical Ethical Committee and the respective participating pharmaceutical companies. The date and the identity of the person responsible of breaking the code should also be documented. (See below and Appendix 2)
It is the investigator' responsibility to ensure that there is a procedure in place to allow access to the code break cards in case of emergency. The investigator will inform the patient how to contact his/her backup in cases of emergency when he/she is unavailable. Study drug must be discontinued after emergency unblinding. Study drug also must be discontinued for any patient whose treatment code has been broken inadvertently or for any non-emergency reason.

SAFETY

All subjects will be monitored as outlined in Appendix 1. After randomization, visits are scheduled at

weeks 2, 8, 16, 24, 32, 40, 51, 52, between week 56-58, 62-63 and at study termination (week 64). Telephone consultation by the investigator is scheduled at week 4. During every visit, a history will be taken, physical examination, including assessment of weight, blood pressure, pulse rate, peripheral edema, and laboratory examinations (see above under study design) will be performed. ECG will be performed at baseline and at week 52.
Special attention will be given to the possible occurrence of hepatic function deterioration (ALT, AST, ALP and bilirubin), fluid retention (weight, physical examination), myopathy (CPK) and renal function/serum potassium. Safety assessment, definition of (serious) adverse events and guidelines for discontinuation of study medication are listed in Appendix 2.

STUDY DRUG DISCONTINUATION / WITHDRAWAL FROM THE STUDY
Study drug must be discontinued for a given patient if the investigator determines that continuing it would result in a significant safety risk for that patient.

After the patient has been entered into the study, his/her treatment will be discontinued if one of or more of the following pertain(s):

1. The occurrence of an adverse event or clinically significant laboratory change or abnormality that, in the judgment of the investigator, warrants discontinuation of treatment.
2. Patient's own decision
3. Non-compliance
4. If type 2 diabetes develops ***and***:
   - FPG > 12.4 mmol/L confirmed by a repeated measurement in the absence of an intercurrent illness, or
   - Symptoms of worsening hyperglycemia (i.e., polyuria, polydipsia, weight loss) in the absence of any intercurrent illness or other incidental circumstances potentially causing deterioration of glucose control.
5. Pregnancy
6. Severe or frequent hypoglycemia (i.e., unexplained hypoglycemic events requiring the assistance of another person to treat or > 3 hypoglycemic events per week).
7. Treatment with prohibited concomitant medications as defined above.
8. Subjects cannot undergo the procedures to measure primary endpoints as outlined in this protocol.
9. Subjects need procedures that are not allowed in the protocol.

In addition to these requirements for study drug discontinuation, the investigator should discontinue study drug for a given patient if, on balance, he thinks that continuation would be detrimental to the patient's well-being. The physician can be guided by the criteria above, but may discontinue patients at any time based on his/her clinical judgment. For any patient withdrawn from the study, an alternative candidate will be selected, in order to ultimately meet the calculated sample size/power.

STUDY COMPLETION AND POST-STUDY TREATMENT
The study will be considered completed for an individual patient when he/she completes the double-blind treatment phase followed by the 12-week off-drug period. The study as a whole will be considered completed when all planned randomized patients have completed the double-blind treatment phase followed by the 12-week off-drug period and the final measurements around week no 64 after randomization.

The investigator must provide follow-up medical care for all patients who are prematurely withdrawn from the study, or must refer them – and those who have finished the study but still need additional care, e.g. because of hypertension and/or diabetes - for appropriate ongoing care.

LABORATORY PROCEDURES (+BLOOD VOLUME)
Laboratory procedures to assess the primary endpoints will be performed at one single laboratory (VUMC, Amsterdam). Thus, insulin, C-peptide and proinsulin will be measured at the VUMC

laboratory. Blood glucose and parameters collected during FU visits will be measured at the 3 separate sites.

Estimated blood volume to be obtained from 1 subject during the 64 week period

**Screening**	**approx. 30 ml**
**Pharmacogenetics (optional)**	**7 ml**
**FU Visits (safety) 14 * 9 =**	**126 ml**
**Clamp test : 97.5 * 3 =**	**292.5 ml**
**OGTT 30 + (70 * 3) =**	**240 ml**
**Markers + spare 30 * 3=**	**90 ml**
**Total blood volume:**	**785.5 ml**

STATISTICAL CONSIDERATIONS

General Considerations

The null hypotheses will be tested at a two-sided significance level of 0.05. For each of the study drugs, rosiglitazone and valsartan, there is a null hypothesis H_0*: No difference exists between the study drug and dummy treatment.* The null hypothesis for each drug will be tested against the hypothesis H_1*: A difference exists between the study drug and dummy treatment, with respect to the underlying parameter of interest*. Using a factorial design, we will test the hypotheses for both study drugs from the complete data set. We assume that the interaction, if any, between the 2 study drugs will be too small to lead to significant deviation of the additive effects of the drugs.

Last observation carried forward (LOCF) method will be used to impute missing efficacy data if necessary; however, baseline values will not be carried forward. A detailed statistical analysis plan will be produced prior to conduct of final data analysis.

ANALYSIS POPULATION

The intent-to-treat population (ITT) will consist of all subjects who took at least one dose of the randomized study medication (rosiglitazone or valsartan) beginning at the day of the randomization. The evaluable population will consist of all ITT subjects who complete the 52-week treatment period in compliance with the protocol.

STUDY ENDPOINTS

Primary Study Endpoints

There are two primary endpoints: 1) the treatment effect on the β-cell function as measured by the ratio of week 52 first phase insulin secretion (the incremental AUC of insulin with respect to basal value over a 10 min period, clamp time 210 min to 220 min) and 2) the treatment effect on the arginine-stimulated insulin secretion during a hyperglycemic clamp, specifically, the incremental AUC of insulin with respect to basal value over a 10 min period (i.e., clamp time 290 min to 300 min) to that at baseline (i.e. clamp performed between week –2 and randomization).

JUSTIFICATION OF SAMPLE SIZE

Sample size calculations are based on the primary efficacy endpoint, the treatment effect on the β-cell function as measured by the ratio of week 52 first phase insulin secretion (the incremental AUC of insulin with respect to basal value over a 10 min period, clamp time 210 min to 220 min) and/or arginine-stimulated insulin secretion during a hyperglycemic clamp, specifically, the incremental AUC of insulin with respect to basal value over a 10 min period (i.e., clamp time 290 min to 300 min) to that at baseline ((i.e. clamp performed between week -2 and randomization).

Similar calculations were performed for people with type 2 diabetes in the ‘Exenatide study’ (protocol 2993-114). In the exenatide study, the effects of a 52-week treatment with exenatide and insulin glargine on similar β-cell function parameters are compared. To summarize, in that study it was assumed that the mean (SD) incremental AUC (iAUC) at baseline is 200 (300 pmol/L·min) for both groups and at week 52, 1100 (1300 pmol/L·min) and 300 (400 pmol/L·min) for subjects treated with exenatide and insulin glargine respectively. Furthermore, it is assumed that iAUC to follow a log-normal distribution. Under these conditions, 26 subjects per group will be needed to provide 90%

power to detect a significant difference between the two groups when tested using independent sample t-test at a 2-sided significance level of 0.05.
In the present study, however, it is unclear a) what the natural course of the β-cell function parameter will be in this study population, b) whether a beneficial effect of the respective study drugs will be found and c) whether this effect will be different for rosiglitazone as compared to valsartan. In the literature, there is no data available with respect to the spontaneous course of the proposed β-cell parameters in subjects fulfilling the proposed inclusion criteria (i.e. those with IFG/IGT and a family history of type 2 diabetes) during one year, nor the effect of the study drugs on these variables. Therefore, the scarce literature data were used and extrapolated to yield a base for sample size calculations.
a) Very recent studies, performed in small groups of DM2 patients for up to 6 months, indicate that several parameters of β-cell function improve after rosiglitazone therapy (58). Ovalle and Bell showed a 43% increase in β-cell function, measured as disposition index derived from C-peptide response to glucagon, after 6 months of 8 mg rosiglitazone treatment in 9 DM2 patients who were inadequately controlled on maximized oral antihyperglycemic therapy. In contrast, addition of (premixed) insulin in the controls group (n=8) resulted in 9.4% decrease in β-cell function (58). Although the natural course of deterioration of the β-cell function over 1 year may be up to 10% in subjects with IFG/IGT (58, 59), we assume a more conservative decline of 5% (60). The above-mentioned study showed that 6 months of rosiglitazone therapy may improve β-cell function by about 40%, however, taken into account the unpredictable influence on the β-cell function ameliorating effect due to a different population, methodology and intervention duration, we assume a more conservative estimation of β-cell function improvement by 25%. Since there are no data available on long-term effects of valsartan (or other ARBs) on β-cell function, we assume that the beneficial effect of valsartan will be similar to that of rosiglitazone (i.e. 25% improvement). Unfortunately, no SD of the percentual changes was stated in the paper.
Since we have defined to co-primary endpoints, adjustment for multiplicity is necessary. To this purpose we will use the Hochberg procedure (61). With this procedure we adjusted the sample size for $\alpha/2 = 2.5$ %.

As stated above, using a 2 x 2 factorial design, and assuming that no interaction exists between the 2 drug regimens – in this case rosiglitazone and valsartan – sample size calculation may be performed as if 2 groups be compared (63). In addition, assuming the above-mentioned conservative changes in β-cell function during the study, the sample size calculation would be as follows: if we assume that there is 5% deterioration of β-cell function over 1 year in the dummy group and 25% improvement in the treated groups (SD = 50%; power=0.8 and significance level = 0.025) than a group of 54 evaluable subjects per group should be sufficient. However, as a safeguard against moderate deviations from the above-mentioned assumptions (i.e. no interactions between the 2 study drugs and equal standard deviations) we increased the calculated sample size by 10%. Finally, we assume a drop-out rate of 20% in this study. Taken together, we will include a total number of approximately 144 subjects (n= (2 x 54) + 20% (22) + 10% (13)).

Number of subjects: Sample size will be approx. **144** (approximately n=36 per treatment arm).

STATISTICAL ANALYSIS

Primary endpoint analyses

Two-way analysis of variance will be used to detect the difference in the co-primary endpoints. The analysis will be stratified by center. Correction will be made for fasting plasma glucose and 2-h postload glucose (as a continuous variable).
Adjustment for multiple testing for the first and the second primary hypotheses will be done using the Hochberg procedure (Hochberg 1988). This procedure strongly controls the multiple significance level of $\alpha=0.05$.

The testing procedure will be as follows: The Hochberg procedure starts with the largest p-value and works according to the following decision rules after ordering of the p-values $p(1) \leq p(2)$ for the two primary hypotheses H(1) and H(2):

Step 1: If $p(2) \leq \alpha$ YES → STOP and reject both null hypotheses

NO → retain H(2) and proceed

Step 2: If $p(1) \leq \alpha/2$ YES → STOP and reject H(1)

NO → retain all null hypotheses

Note : As stated above, with this procedure we adjusted the sample size for $\alpha/2 = 2.5$ % for p(1)

Secondary endpoint analyses
Differences in continuous variables will be tested by two-way ANOVA. Differences in binary variables will be examined by logistic regression.
Although we assume no interaction, we will test for interaction between the 2 study drugs by using two-way ANOVA. It should, however, be kept in mind that testing for interaction have less power than tests for the main effects. Separate subgroups analyses will be made for gender, IFG, IGT and IFG + IGT. Also here, there will be less power to detect differences than in the tests for main effects.

SUBSTUDIES
There will be 3 official sub-studies to the PRESERVE protocol, i.e. **sub-study 1**) Assessment of rosiglitazone and valsartan effects on abdominal fat distribution and liver fat accumulation in relation to β-cell function changes; **sub-study 2**) Assessment of rosiglitazone and valsartan effects on fatty acid partitioning, body fat distribution and molecular mechanisms in fat and muscle; **sub-study 3**) Assessment of rosiglitazone and valsartan effects on carotid IMT/stiffness and microvascular function.

ETHICS & LEGAL REQUIREMENTS
The general principles of informed consent, ethics review and data management will be in line with GCP. The study will be conducted in accordance with the principles stated in the Declaration of Helsinki.

REFERENCES

1. DeFronzo RA. Pathogenesis of type 2 (non-insulin-dependent) diabetes mellitus: a balanced overview. Diabetologia 1992;35:389-97
2. Yki-Jarvinen H. Pathogenesis of non-insulin-dependent diabetes mellitus. Lancet 1994;343:91-5
3. Bailey CJ. Potential new treatments for type 2 diabetes. Trends Pharmacol Sci 2000;21:259-65
4. Dubois M, Pattou F, Kerr-Conte J, et al. Expression of PPARγ in normal human pancreatic islet cells. Diabetologia 2000;43:1165-9
5. Zhou YT, Shimabukuro M, Wang MY, et al. The role of PPARα in disease of pancreatic β cells. Proc Natl Acad Sci USA 1998;95:8898-8903
6. Higa M, Zhou YT, Ravazzola M, et al. Troglitazone prevents mitochondrial alterations, β cell destruction, and diabetes in obese prediabetic rats. Proc Natl Acad Sci USA 1999;96:11513-8
7. Kim H, Ahn Y. Role of PPARγ in the glucose-sensing apparatus of liver and β-cells. Diabetes 2004;53(S1):S60-S65
8. Shimabukuro M, Zhou YT, Lee Y, et al. Troglitazone lowers islet fat and restores beta cell function in ZDF rats. J Biol Chem 1998;273:3547-50
9. Koh EH, Kim MS, Park JY, et al. PPARα activation prevents diabetes in OLETF rats: comparison with PPARγ activation. Diabetes 2003;52:2331-7
10. Kersten S, Desvergne B, Wahli W. Roles of PPARs in health and disease. Nature 2000;405:421-4
11. Rosen ED, Kulkarni RN, Ozcan U, et al. Targeted elimination of PPARγ in β cells leads to abnormalities in islet mass without compromising glucose homeostasis. Mol Cell Biol 2003;23:7222-9
12. Strazzullo P, Galletti F. Impact of the renin-angiotensin system on lipid and carbohydrate metabolism. Curr Opin Nephrol Hypertens 2004;13(3):325-32
13. Unger T. The role of the renin-angiotensin system in the development of cardiovascular disease. Am J Cardiol 2002;89(2A):3A-9A

14. Strawn WB, Ferrario CM. Mechanisms linking angiotensin II and atherogenesis. Curr Opin Lipidol 2002;13:505-12
15. Folli F, Kahn CR, Hansen H, et al. Angiotensin II inhibits insulin signaling in aortic smooth muscle cells at multiple levels. A potential role for serine phosphorylation in insulin/angiotensin II crosstalk. J Clin Invest. 1997;100:2158-69
16. Bernobich E, de Angelis L, Lerin C, et al. The role of the angiotensin system in cardiac glucose homeostasis: therapeutic implications. Drugs 2002;62:1295-314
17. Nascimben L, et al. J Hypertension 1997;15(S4):S84
18. Lau T, Carlsson PO, Leung PS. Evidence for a local angiotensin-generating system and dose-dependent inhibition of glucose-stimulated insulin release by angiotensin II in isolated pancreatic islets. Diabetologia 2004;47:240-8
19. Bergman RN, Finegood DT, Kahn SE. The evolution of beta-cell dysfunction and insulin resistance in type 2 diabetes. Eur J Clin Invest 2002;32 Suppl 3:35-45
20. Tikellis C, Wookey PJ, Candido R, et al. Improved islet morphology after blockade of the renin- angiotensin system in the ZDF rat. Diabetes 2004;53(4):989-97
21. Yusuf S, Gerstein H, Hoogwerf B, et al. Ramipril and the development of diabetes. JAMA. 2001 Oct 17;286(15):1882-5.
22. Lindholm LH, Ibsen H, Borch-Johnsen K, et al. Risk of new-onset diabetes in the Losartan Intervention For Endpoint reduction in hypertension study. J Hypertens. 2002 Sep;20(9):1879-86
23. Niklason A, Hedner T, Niskanen L, et al. Development of diabetes is retarded by ACE inhibition in hypertensive patients--a subanalysis of the Captopril Prevention Project (CAPPP). J Hypertens. 2004 Mar;22(3):645-52
24. Devereux RB, Dahlof B, Kjeldsen SE, et al. Effects of losartan or atenolol in hypertensive patients without clinically evident vascular disease: a substudy of the LIFE randomized trial. Ann Intern Med. 2003 Aug 5;139(3):169-77
25. Lithell H, Hansson L, Skoog I, et al. The Study on Cognition and Prognosis in the Elderly (SCOPE): principal results of a randomized double-blind intervention trial. J Hypertens. 2003 May;21(5):875
26. Scheen AJ. Prevention of type 2 diabetes mellitus through inhibition of the Renin-Angiotensin system. Drugs 2004;64:2537-65
27. Julius S, Kjeldsen SE, Weber M, et al. Outcomes in hypertensive patients at high cardiovascular risk treated with regimens based on valsartan or amlodipine: the VALUE randomised trial. Lancet 2004;363:2022-31
28. Diamant M, Heine RJ. Thiazolidinediones in type 2 diabetes mellitus: current clinical evidence. Drugs 2003;63: 1373-405
29. Olefsky JM. Treatment of insulin resistance with peroxisome proliferator-activated receptor γ agonists. J Clin Invest 2000;106:467-72
30. Schoonjans K, Auwerx J. Thiazolidinediones: an update. Lancet 2000;355:1008-10
31. Martens FM, Visseren FLJ, Lemay J, et al. Metabolic and additional vascular effects of thiazolidinediones. Drugs 2002;62:1463-80
32. Nolan JJ, Ludvik B, Beerdsen P, et al. Improvement in glucose tolerance and insulin resistance in obese subjects treated with troglitazone. N Engl J Med 1994;331:1188-93
33. Cavaghan MK, Ehrmann DA, Byrne MM, et al. Treatment with the oral antidiabetic agent troglitazone improves beta cell responses to glucose in subjects with impaired glucose tolerance. J Clin Invest 1997;100:530-7
34. Buchanan T, Xiang AH, Peters RK, et al. Preservation of Pancreatic β-cell function and prevention of type 2 diabetes by pharmacological treatment of insulin resistance in high-risk Hispanic women. Diabetes 2002;51:2796-2803
35. Diep QN, El Mabrouk M, Cohn JS, et al. Structure, endothelial function, cell growth, and inflammation in blood vessels of angiotensin II-infused rats: role of peroxisome proliferator-activated receptor-gamma. Circulation 2002;105:2296-302
36. DeFronzo RA, Tobin JD, Andres R. Glucose clamp technique: a method for quantifying insulin secretion and resistance. Am J Physiol 1979;237:E214-E223
37. Bardet S, Pasqual C, Maugendre D, et al. Inter and intra individual variability of acute insulin response during intravenous glucose tolerance tests. Diabetes Metab 1989;15:224-232
38. Hosker JP, Matthews DR, Rudenski AS, et al. Continuous infusion of glucose with model assessment: measurement of insulin resistance and βcell function in man. Diabetologia 1985;28;401-11
39. Scheen AJ, Castillo MJ, Lefebvre PJ. Assessment of residual insulin secretion in diabetic patients using the intravenous glucagons stimulatory test: methodological aspects and clinical applications. Diabetes Metab 1996;22:397-406
40. Pacini G, Mari A. Methods for clinical assessment of insulin sensitivity and β-cell function. Best Pract Res Clin Endocrinol Metab. 2003 Sep;17(3):305-22
41. Larsson H, Ahrén B. Glucose-dependent arginine stimulation test for characterization of islet function: studies on reproducibility and priming effect of argnine. Diabetologia 1998;41:772-7
42. Fritsche A, Stefan N, Hardt E, et al. Characterisation of β-cell dysfunction of impaired glucose tolerance: evidence for impairment of incretin-induced insulin secretion. Diabetologia 2000;43:852-8
43. Mari A, Schmitz O, Gastaldelli A, et al. Meal and oral glucose tests for assessment of β-cell function: modelling analysis in normal subjects. Am J Physiol 2002;283:E1159-E1166
44. Executive Summary of the Third Report of the National Cholesterol Education Program (NCEP) Expert Panel on Detection, Evaluation, and Treatment of High Blood Cholesterol in Adults (ATPIII). JAMA 2001;285:2486-97
45. Lorenzo C, Okoloise M, Williams K, et al. The metabolic syndrome as predictor of type 2 diabetes: the San Antonio Heart Study. Diabetes Care 2003;26:3153-9
46. Laaksonen DE, Lakka HM, Niskanen LK, et al. Metabolic syndrome and development of diabetes mellitus: application and validation of recently suggested definitions of the metabolic syndrome in a prospective cohort study. Am J Epidemiol 2002;156:1070-7

46a. Dalla Man C, Campioni M, Polonsky KS, et al. Two-hour seven-sample oral glucose tolerance test and meal protocol: minimal model assessment of β-cell responsivity and insulin sensitivity in nondiabetic subjects. Diabetes

2005;54:3265-73

47. Marchesini G, Forlani G, Cerrelli F, et al. WHO and ATPIII proposals for the definition of the metabolic syndrome in patients with type 2 diabetes. Diabet Med 2004;21:383-7
48. Isomaa B, Henricsson M, Almgren P, et al. The metabolic syndrome influences the risk of chronic complications in patients with type II diabetes. Diabetologia 2001;44:1148-54
49. Ehrmann DA, Schneider DJ, Sobel BE, et al. Troglitazone improves defects in insulin action, insulin secretion, ovarian steroidogenesis, and fibrinolysis in women with PCOS. J Clin Endocrinol Metab 1997;82:2108-16
50. Alexander CM, Landsman PB, Teutsch SM, et al. Third National Health and Nutrition Examination Survey (NHANES III); National Cholesterol Education Program (NCEP). NCEP-defined metabolic syndrome, diabetes, and prevalence of coronary heart disease among NHANES III participants age 50 years and older. Diabetes 2003;52:1210-4
51. Sattar N, Gaw A, Scherbakova O, et al. Metabolic syndrome with and without C-reactive protein as a predictor of coronary heart disease and diabetes in the West of Scotland Coronary Prevention Study. Circulation 2003;108:414-9
52. Festa A, D'Agostino R Jr, Howard G, et al. Chronic subclinical inflammation as part of the insulin resistance syndrome: the Insulin Resistance Atherosclerosis Study (IRAS). Circulation 2000;102:42-7
53. Haffner SM, Greenberg AS, Weston WM, et al. Effect of rosiglitazone treatment on nontraditional markers of cardiovascular disease in patients with type 2 diabetes mellitus. Circulation 2002;106:679-84
54. Wang TD, Chen WJ, Lin JW, et al. Effects of rosiglitazone on endothelial function, C-reactive protein, and components of the metabolic syndrome in nondiabetic patients with the metabolic syndrome. Am J Cardiol 2004;93:362-5
55. Watanabe Y, Sunayama S, Shimada K, et al. Troglitazone improves endothelial dysfunction in patients with insulin resistance. J Atheroscler Thromb 2000;7:159-63
56. Caballero AE, Saouaf R, Lim SC, et al. The effects of troglitazone, an insulin-sensitizing agent, on the endothelial function in early and late type 2 diabetes: a placebo-controlled randomized clinical trial. Metabolism 2003;52:173-80
57. Prisant LM. Preventing type II diabetes mellitus. J Clin Pharmacol 2004;44(4):406-13
58. Ovalle F, Bell DSH. Effect of rosiglitazone versus insulin on the pancreatic β-cell function of subjects with type 2 diabetes. Diabetes Care 2004;27 :2585-9
59. Matthews DR, Cull CA, Stratton IM, et al . UKPDS 26: Sulphonylurea failure in non-insulin-dependent diabetic patients over six years. UK Prospective Diabetes Study (UKPDS) Group. Diabet Med 1998;15:297-303
60. Walker M, Mari A, Jayapaul MK, et al. Impaired beta cell glucose sensitivity and whole-body insulin sensitivity as predictors of hyperglycaemia in non-diabetes subjects. Diabetologia 2005;48:2470-6
61. Hochberg Y. A sharper Bonferroni procedure for multiple tests of significance. Biometrika 1988 ;75 :800-2
62. Padwal R, Mamdani M, Alter DA, et al. Antihypertensive therapy and incidence of type 2 diabetes in an elderly cohort. Diabetes Care 2004 ;27 :2458-63
63. Fleiss JL. Design and analysis of clinical trials. Wiley New York, 1986

Appendix 1: Study Plan
PRESERVE PROTOCOL

**Visit**	**1**	**2**	**3**	**4**	**5**	**T1**	**6**	**7**	**8**	**9**	**10**	**11**	**12**	**13**	**14**	**Study Term 15**	**Early Term**
**Week Relative to Randomization**	**Screen**	**-2**	**-1**	**Day 1**	**2**	**4**	**8**	**16**	**24**	**32**	**40**	**51**	**52**	**56/58**	**62/63**	**64**	
Informed Consent	X																
Medical History/Height	X																
Questionnaires	X																
Vital Signs/Anthropometrics[a]	X	X	X	X	X		X	X	X	X	X	X	X			X	X
Physical Examination/12-lead ECG	X												X	X			X[b]
Pregnancy Test (βhCG)[c]	X			X												X	X
HbA_{1c}/Fasting Plasma Glucose/Lipids	X			X			X	X	X	X	X		X	X		X	X
Chemistry, Hematology	X			X	X		X	X	X	X	X		X			X	X
Urinalysis	X			X			X		X				X			X	
HOMA-IR	X			X									X			X	
Pharmacogenetics Sample[d]	X																
Adverse Event/Medications Review		X	X	X	X	X	X	X	X	X	X		X	X	X	X	X
Study-Related Training[e]																	
Randomization				X													
Study Medication Titration					X												
Study Medication Dosing Period																	
Study Medication Dispensed				X	X		X	X	X	X	X						
Collect Used/Unused Study Medication					X		X	X	X	X	X		X				X
24-h Urine Collection/Return			X										X				X
Oral Glucose Tolerance Test	X[f]		X[g]									X[g]			X[g]		X
Circulating Markers[h]			X									X				X	X
Euglycemic/Hyperglycemic Clamp Tests		X											X			X	
**Carotid artery IMT/DC Measurement**[i]			**X**									**X**					
**MRI/^{1}H-MRS** [j]		**X**											**X**				
**Fatty acid partitioning/biopsies** [k]		**X**[k]	**X**[k]									**X**[k]	**X**[k]				
**Microvascular measurements**			**X**									**X**					

[a] Anthropometrics: Height measured at baseline only; blood pressure, heart rate and body weight will be measured at all visits indicated, waist circumference at baseline, 24, 52 and 64 weeks
T1: adverse events/medication review by telephone
[b]Performed only if study withdrawal occurs prior to Visit 13 (Week 52).
[c] Pregnancy test performed only in females of child-bearing potential; at baseline and at week 52 (Visit 13)
[d] Pharmacogenetic blood samples are optional and should only be collected after the pharmacogenetic informed consent is signed
[e] Education / instruction regarding life style and possible adverse events of the study medications will be provided to all participants.
[f] The 75-g OGTT at screening will be used to classify subjects into glucose tolerance categories, only blood samples at t=0 and t=2 h will be collected.
[g] After inclusion, 75-g OGTT will be performed and blood samples will be collected at t=0, 10, 20, 30, 60, 90 and 120 min and stored at –80C until analysis.
Blood glucose values obtained from non-screening OGTT will not be used for classification purposes.
[h] Markers include: markers of inflammation, adipokines (leptin, adiponectin), and additional samples will be collected for potential new markers pertinent to the study
[i,j] Procedures for substudies 3 and 1, respectively,
[k] **Substudy 2: At Maastricht fatty acid partitioning / adipose tissue and muscle biopsies will be performed during separate visits, between wk –4 – day 1, and at 50-52 weeks. At VUMC, biopsies will be taken at visit 3 and 11**

Appendix 2: Safety Considerations And Reporting PRESERVE PPROTOCOL

INTRODUCTION
The PRESERVE trial is an investigator initiated trial and will be performed by investigators of the VU University Medical Center, Amsterdam, The Netherlands. The trial is funded by GSK and Novartis.
The study will be performed at 3 locations, i.e. The VU University Medical Center, Amsterdam, the Hoorn Research Center in Hoorn (affiliated to the VUMC and directed by dr. G. Nijpels) and Department of Human Biology, Nutrition Research Center, Maastricht University, Maastricht, The Netherlands (supervised by dr. E.E. Blaak). At each location approximately 48 subjects will be studied.
This document outlines the definitions and procedures for reporting safety data in this study, the responsibilities of the different parties, and the arrangements for conduct and cooperation between the parties in respect of pharmacovigilance.

BACKGROUND
As noted in the protocol, approximately 144 participants with IFG (with/without a family history of DM2) and/or IGT will be randomly allocated to either rosiglitazone (RSG) or valsartan (VAL) using a 2X2 factorial design and followed for up 52 weeks after randomization while on treatment and subsequently for another 12 weeks afdter discontinuation of study medication. Participants will be assessed at regular intervals to ascertain safety and tolerability as well as compliance. Primary outcome measures (i.e. β-cell function assessed by both clamp and OGTT) and other secondary outcomes (anthropometric, metabolic and vascular variables as well as markers of inflammation) will be assessed before and at approximately 52 and 64 weeks following randomization. Both RSG and VAL have been prescribed to millions of patients all over the world. Nevertheless, as for RSG, regulatory authorities require that all patients given any thiazolidendiones undergo systematic hepatic enzyme monitoring especially during the first year of therapy; this is clearly written into the PRESERVE protocol. With regard to VAL, regular assessment of blood pressure and serum potassium and creatinine is advocated.

Definitions and Procedures for Reporting Adverse Events
The PRESERVE trial is studying individuals with IFG with or without a family history of DM2 and/or IGT with/ without the metabolic syndrome who are at high risk for both diabetes and cardiovascular disease (CVD) events (including death due to cardiovascular causes) compared to individuals with normal glucose tolerance. Although the development of diabetes and/or cardiovascular disease are not the primary efficacy variable in this protocol, the occurrence of these events should be recorded in detail. Depending on its severity (see below) CVD should be regarded as a (serious) adverse event (AE). Detailed information describing all (S)AEs will be collected on specific case report forms and sent to Novartis (see below)

Definition of a Serious Adverse Event
Any untoward medical occurrence that at any dose:

- Results in death
- Is life-threatening
- Requires inpatient hospitalisation or prolongation of existing hospitalisation
- Results in persistent or significant disability/incapacity
- Is a congenital anomaly/birth defect
- Is an 'other' significant event.

Reporting responsibility

Each serious adverse event must be reported by the investigator to Novartis within 24 hours of learning of its occurrence, even if it is not felt to be treatment-related. Follow-up information about a previously reported serious adverse event must also be reported within 24 hours of the investigator receiving it. If the serious adverse event is not previously documented (new occurrence) and is thought to be related to the Novartis/GSK study drug (or therapy), a Clinical Safety & Epidemiology Department associate may urgently require further information from the investigator for Health Authority reporting. Novartis/GSK may need to issue an investigator notification, to inform all investigators involved in any study with the same drug (or therapy) that this serious adverse event has been reported.

Reporting procedures

The investigator must complete the Serious Adverse Event Report Form in English, assess the relationship to study treatment and send the completed, signed form by fax within 24 hours to the local Novartis Clinical Safety & Epidemiology Department (for trials monitored by Novartis). The original copy of the Serious Adverse Event Form and the fax confirmation sheet must be kept with the case report form documentation at the study site.

Follow-up information is sent to the same person sent the original Serious Adverse Event Form. A new serious adverse event form is sent, stating that this is a follow-up to the previously reported serious adverse event and giving the date of the original report. Each re-occurrence, complication or progression of the original event should be reported as a follow-up to that event. The follow-up information should describe whether the event has resolved or continues, if and how it was treated, whether the blind was broken or not, and whether the patient continued or discontinued study participation. The form and fax confirmation sheet must be retained. Refer to the Novartis guidelines as needed for instructions for completing the Serious Adverse Event Form.

Contact persons and numbers

The telephone and telefax numbers of the contact persons in the participating departments of the 3 sites, are listed below:

VUMC, Amsterdam: dr. (study physician) / Dr. M. Diamant / prof. Dr. R.J. Heine, Department of Endocrinology/Diabetes Center, VU University Medical Center; Phone: +31-20-4440533/Fax +31-20-4440502

Department of Pharmacy, VUMC, Amsterdam: Dr. P. Bet; phone +31-20-4443546 / +31-20-4444444 (tracer *986703)

Hoorn Research Center, Hoorn: (study physician) / Dr. G. Nijpels; phone: +31-20-4449659
MU, Maastricht: Dr. (study physician) / Dr. E. Blaak; phone: +31-43-3881503 / fax: +31-43-3670976

Instructions for rapid notification of pregnancies

Each pregnancy that started during the study must be reported by the investigator to Novartis within 24 hours of learning of its occurrence.

Emergency procedure for unblinding

Emergency unblinding should only be done when necessary in order to treat the patient. Most often, study drug discontinuation and knowledge of the possible treatment assignments are sufficient to treat a study patient who presents with an emergency condition. Coded and sealed envelopes for each participant will be stored in a place where they are readily available to the study physician in case of a serious adverse event (SAE). In the case of a SAE or a medical emergency requiring knowledge of the study medication, the study physician will break the treatment code by opening the envelope. When the investigator breaks the code, he/she must note the date, time, and reason for removing it and retain this information with the case report form documentation. Reasons for breaking the code will be explained clearly and justified in writing to the Medical Ethical Committee and the respective participating pharmaceutical companies. The date and the identity of the person responsible of breaking the code should also be documented. It is the investigator' responsibility to ensure that there is a procedure in place to allow access to the code break cards in case of emergency. The investigator will inform the patient how to contact his/her backup in cases of emergency when he/she is unavailable. Study drug must be discontinued after emergency unblinding. Study drug also must be discontinued for any patient whose treatment code has been broken inadvertently or for any non-emergency reason.

FAXNUMBER FOR THE SAE FORMS: 026-3782414

Pauline Pernot, M.D.	*Marlies Wanningen*	*Novartis Pharma B.V.*
Clinical Safety Officer	*CS&E secretary*	*Raapopseweg 1*
Tel: + 31 26 3782243	*Tel +31 26 3782185*	*6824 DP Arnhem*

Appendix 3:
Euglycemic and Hyperglycemic Clamp Procedure with Arginine Stimulation
PRESERVE PROTOCOL

The total study population, approximately 144 subjects (36 in each treatment arm) will undergo the clamp procedure at Visit 2 (Week -2), Visit 11 (Week 51) and Visit 14 (Week 63).

Preparation of subjects. The subjects will arrive at the metabolic ward in the morning after an overnight fast (at least 8 h). All subjects will remain fasted throughout the clamp. At the evening prior to the clamp procedure during Visit 11 (Week 51), subjects will be instructed to take their usual medication (e.g. statins, blood pressure lowering drugs) as well as their study medication at bed-time. They should refrain from taking any drugs in the morning of the clamp day. All subjects will resume their medications, including the study medication, after finishing the clamp procedure.

Subjects will be placed in a semirecumbent position, and a cannula will be inserted into the non-dominant (if available) antecubital fossa for infusion of saline, glucose, and insulin. A contralateral dorsal hand vein will be cannulated in a retrograde manner and maintained at 55º C to permit sampling of arterialized venous blood at 5 min intervals throughout the clamp protocol. The cannulae will be kept patent 0using 0.9% saline (saline in a 2 mL syringe; no heparin will be used). After both cannulae have been inserted, the veins will be allowed at least 15 min of rest time prior to starting blood draws for the clamp.

Glucose (20%) will be infused at a variable rate to maintain glucose at the desired concentration. The glucose infusion rate will be adjusted according to bedside measurement of blood glucose (BG) every 5 min based on a predefined algorithm. Blood samples will be taken before and during the tests as described in the following table. In all subjects, a euglycemic insulin clamp will be performed during the first 2 h followed by an interval ("rest period") of 1 hour during which insulin infusion will be discontinued and glucose concentration maintained by the clamp technique, and a subsequent hyperglycemic clamp procedure with a standard 80-min square-wave hyperglycemia of 15 mmol/L. During the last 30 min of the hyperglycemic clamp, an arginine bolus will be administered. The duration of the total procedure is about 380 min (6 h, 20 min), during which a total amount of approximately 97.5 mL blood will be collected.

The euglycemic clamp procedure. The induction phase of the clamp protocols will start at 0800. Each insulin infusion will be primed with an intravenous (IV) insulin bolus of 0.1 x kg body weight x desired elevation of plasma insulin level of 100 mU/L. The insulin infusion rate will be maintained at 40 $mU/min/m^2$ body surface area (compatible with 0.24 $nmol/min/m^2$) during the 2-h euglycemic clamp. The blood glucose concentration will be maintained at around 5 mmol/L by performing manual glucose clamp for the remaining duration of the euglycemic clamp procedure. The glucose infusion rates will be adjusted according to the equation originally described by DeFronzo et al. (1979) with supplementary glucose infusion when BG decreases by > 0.2mmol/L in 5 min. The blood glucose concentration reached at a maximum of 90 min after onset of the clamp procedure, should be regarded as the clamp target blood glucose concentration at which blood glucose concentration should be kept during the following, final 30 min of the clamp.

Resting period. During the subsequent resting period of 60 min the subjects will receive an infusion of saline or glucose to maintain euglycemic levels at around 5 mmol/L (or at the blood glucose concentration reached at 90 min of the euglycemic clamp). Insulin infusion will be discontinued during this period.

The hyperglycemic clamp procedure combined with arginine stimulation. The hyperglycemic clamp procedure will be started with a body weight adjusted IV bolus of 20% glucose administered over 1 min followed by a continuous infusion to achieve target blood glucose concentration of approximately 15 mmol/L (27 x bodyweight [kg] x desired increase in blood glucose [mmol/L] = bolus dose [mg]). The glucose infusion will be adjusted to maintain BG at 15 mmol/L. After 80 min, an IV bolus of 5 g arginine (dissolved in 50 mL) will be injected over 45 s while the glucose level will be maintained at 15 mmol/L.

Blood collections and sample handling. Blood glucose concentrations will be determined by the glucose oxidase method with a Yellow Springs glucose analyzer (Yellow Springs, OH, US) at 5 min intervals, except for the first 10 min of the hyperglycemic clamp (at 3 min, 5 min, 8 min, and 10 min) and the first 5 min after the arginine bolus (at 2 min, 3 min, 4 min, and 5 min). Sampling for plasma insulin, C-peptide, proinsulin and glucagon and will be performed as in the following table. All samples will be processed according to standard operating procedures within 1 h and stored at -20°C until assay.

Euglycemic and Hyperglycemic Clamp Procedures

Test Time (min)	Real time (min)	Procedures	Period	Analytes: Insulin	C-Peptide	Proinsulin	Glucagon	Blood Volume (mL)
-30	0			X	X		X	3.5
-15				X	X		X	3.5
0*	30	Begin insulin infusion	Euglycemic Clamp					
60								
90		Reach glucose clamp target (5 mM)		X	X	X	X	3.5
120	150	End insulin infusion	Rest Period	X	X	X	X	3.5
-10 (=50)				X	X	X		1.5
-5 (=55)				X	X	X		1.5
0 (=60)	210	Begin glucose infusion (target 15 mM)	Hyperglycemic Clamp					
1				X	X			1.5
2				X	X			1.5
3*				X	X	X		1.5
4				X	X			1.5
5				X	X	X		1.5
6				X	X			1.5
7				X	X			1.5
8*				X	X			1.5
9				X	X			1.5
10				X	X	X		1.5
15				X	X			1.5
30				X	X	X		1.5
60				X	X	X		1.5
70				X	X			1.5
80	290	I.V. Arginine Bolus (45s)	Arginine Challenge	X	X			1.5
81				X	X			1.5
82*				X	X			1.5
83*				X	X			1.5
84*				X	X			1.5
85				X	X			1.5
86				X	X			1.5
87				X	X			1.5
88				X	X			1.5
89				X	X			1.5
90				X	X			1.5
100				X	X			1.5
110	320	End of Clamp	Recovery Period	X	X			1.5
170	380	End Recovery						
**Subtotal Blood Volume (mL)**								**57.5**
***Blood Volume for Glucose Analysis**								**40**
**Total Blood Volume (mL)**								**97.5**

BG, blood glucose; YS, Yellow Springs glucose analyzer
*Assessments of BG/YS are performed every 5 min, and at time points indicated by an asterisk.
Rest period: if BG starts to rise <60 min, the hyperglycemic clamp should be started earlier.
Number of assessments per clamp procedure: insulin (33), c-peptide (33), proinsulin (9), and glucagon (4)

Appendix 4
Oral Glucose Tolerance Test
PRESERVE PROTOCOL

75 gram Oral Glucose Tolerance Test (OGTT)
<u>OGTT at screening</u>

1. At screening a standardized 75-g OGTT will be performed after an overnight fast. Subjects will be instructed to drink 100 ml of glucose solution (75 g) within 60 sec. Before and 120 min h after administration of the glucose load, blood will be collected for determination of glucose, insulin, C-peptide (total volume max 10 ml).

<u>OGTT at visits 3, 11 and 15</u>
At visit 3 (week –1), visit 12 (week 52) and visit 15 (week 64) an adapted 75-g OGTT will be performed after an overnight fast. Subjects will be instructed to drink 100 ml of glucose solution (75 g) within 60 sec. Before and at 10, 20, 30 , 60 , 90 and 120 min h after administration of the glucose load, blood will be collected for determination of glucose, insulin, C-peptide, GLP1, GIP, ghrelin and PYY (total volume max70 ml).

Ref erence
Dalla Man C, Campioni M, Polonsky KS, et al. Two-hour seven-sample oral glucose tolerance test and meal protocol: minimal model assessment of β-cell responsivity and insulin sensitivity in nondiabetic subjects. Diabetes 2005;54:3265-73

Appendix 5
Sub-Study 1
Body fat distribution/liver fat content assessed by MRI/^{1}H-MRS
PRESERVE PROTOCOL

Background:
Liver fat accumulation or steatosis, also termed nonalcoholic fatty liver disease (NAFLD) has gained much interest. NALFD, is an important factor in the pathogenesis of insulin resistance and type 2 diabetes mellitus (DM2). Several studies have found NAFLD to be associated with features of the metabolic syndrome, including hyperinsulinemia, dyslipidemia, central obesity and glucose intolerance, independently of body weight. Currently, NALFD is considered as the hepatic component of the so-called metabolic syndrome and various treatment modalities have been devised aiming to reduce the amount of liver fat and associated cardiovascular (CVD) risk factors. Occasionally, NAFLD can progress to the more severe condition termed non-alcoholic steatohepatitis (NASH).
Treatment with thiazolidinediones (TZD) and more recently, also with angiotensin-receptor antagonists was shown to ameliorate NAFLD and NASH. In particular, TZD lowered liver fat content, increased insulin clearance, improved hepatic insulin resistance and increased circulating adiponectin levels. In patients with NASH, ARB decreased circulating markers of hepatic fibrosis and inflammation and improved histological fibrosis and inflammation.
Thus, it is feasible that therapeutic targeting of NAFLD/NASH will prove an effective strategy to improve the key pathogenic lesion in insulin resistance / type 2 diabetes and the associated CVD risk factors.
Recently, several mechanisms have been described linking central obesity and in particular hepatic steatosis to β-cell dysfunction (9, 10). Thus, the GPR40 receptor on β-cells, which is activated by fatty acids, was proposed to link obesity to the insulin secretory defects in type 2 diabetes. Also, hypoadiponectinemia and high levels of circulating proinflammatory markers - some of which are derived from visceral adipose tissue and/or the fatty liver - may play a role in the development of β-cell dysfunction. However, it is not clear whether abdominal visceral fat and/or liver fat is causally related to alterations in β-cell function.
Liver fat accumulation can be quantified by non-invasive proton magnetic resonance spectroscopy (^{1}H-MRS). This method was extensively validated against lipid content of liver biopsies in humans and animals and successfully used not only in cross-sectional assessments of liver fat but also in follow-up studies after drug intervention.

Objective:
Primary
To assess the changes in abdominal fat distribution and liver fat content, measured by MRI and ^{1}H-MRS, respectively, from baseline after a 52-week treatment with a thiazolidinedione, rosiglitazone (RSG) and an angiotensin-receptor blocker, valsartan (VAL) in patients with IFG and IGT, using a 2 x 2 factorial design within the PRESERVE study.
Secondary
1. To measure treatment-induced changes in liver enzymes (alanine aminotransferase (ALT), aspartate aminotransferase (AST) and γ-glutamyltranspeptidase (GGT)
2. To assess the association between treatment-induced changes in liver fat and parameters of β-cell function

For study design, subjects and follow up, please see the PRESERVE study. MRI/H1-MRS measurements will be performed at Visit 2 (Week -2) and Visit 11 (Week 51) prior to blood collection.

Methods
Abdominal fat depots (visceral, subcutaneous) will be measured by MR imaging according to a previously described protocol at the level of the umbilicus in a 1.5 T MR device (11).
Localized single voxel (2.5 x 2.5 x 2.5 cm^3) proton spectra will be acquired using a body-coil for RF-transmission and spine and body-array coils for signal receiving and a 1.5-T whole-body system (Sonata; Siemens, Erlangen, Germany). Steady-state free precession (SSFP) MR images are employed for localization of the voxel of interest within the right or left lobe of the liver. Major blood vessels, intra-hepatic bile ducts and the lateral margin of the liver are avoided while localizing the voxel.

Subjects are measured in the supine position with the body-array coil positioned at the liver region. The single voxel spectra are recorded by using the stimulated-echo acquisition mode sequence with an echo time of 20 ms, a repetition time of 5,000 ms, and a mixing time of 30 ms. A total of 8 acquisitions will be obtained, and stored and processed for each coil element separately (yielding typically 24 to 40 spectra – depending on the number of coil elements selected). The short echo time and long repetition time are chosen to ensure a fully relaxed water signal, which is used as an internal standard. Chemical shifts are measured relative to water at 4.70 ppm. The methylene and methyl signals, which represent intracellular triglyceride content, are measured at 1.3 ppm and 0.9 ppm, respectively. The spectra are quantified with LCModel (Magn. Reson. Med 1993;30:672-9). After processing the individual spectra, the corresponding phase-corrected raw data are added (taking into account the sensitivity per coil element) yielding one single raw dataset per VOI location. Subsequently, this dataset is analyzed and was quantified again. Spectroscopic intracellular triglyceride content is expressed as the percentage of the area under the methyl and methylene peak relative to that under the water peak.

Sample size calculation: A previous study, evaluating the effect of 16-week RSG therapy on liver fat, measured by ^{1}H-MRS, in DM2 subjects, used a parallel design (RSG versus metformin; 7). These authors detected a significant 51% decrease in liver fat from baseline (from 15 (SD3)% vs 7 (1)%; p=0.003) in n=9 subjects treated with RSG, whereas metformin had no effect on liver fat. The detection of such a significant effect of RSG on liver fat confirms that MRI/^{1}H-MRS are sensitive and reproducible state-of-the art techniques.
However, the current study differs, with respect to population (pre-diabetic) and duration of intervention. Moreover, there are no studies available that measured changes in liver fat by ^{1}H-MRS after ARB treatment in a comparable population. It is conceivable that patients with impaired glucose metabolism, but without diabetes, may have less liver fat at baseline and that a lower hepatic fat content will result in less changes after intervention. Our own data, i.e. obtained from measurements of liver fat in healthy middle aged men, in age-matched men with the metabolic syndrome and in men with DM2, show mean liver fat of 5.4 (SD 6.7)%, 10.5 (10.9)% and 21.2 (17.7)% in these groups, respectively. Based on these findings, we assume that liver fat in the PRESERVE population will range between 5-10%. In order to detect significant therapy-induced changes, the number of subjects to be studied should be higher than that in ref. no 7. Finally, more subjects should be studied in order to reliably calculate an association between changes in abdominal fat depots/ liver fat and treatment-induced alterations in the variables of β-cell function, ánd allowing additional correction for possible confounders.
As in the main protocol of the PRESERVE study, assuming no interaction between the study drugs, we may compare the treatment arms as if a parallel group design (i.e. 2 groups) was employed. Thus, a total of 80 patients (n=40 in Amsterdam and n=40 in Maastricht), may be expected to yield meaningful results and also for correction for possible confounders (e.g. age, gender, BMI, blood glucose, lipids, CRP).

References :

1. Yki-Jarvinen H, Westerbacka J. The fatty liver and insulin resistance. Curr Mol Med 2005;5:287-95
2. Schindhelm RK, Diamant M, Tushuizen ME, et al. Liver enzymes and fatty liver disease in epidemiological studies in relation to type 2 diabetes mellitus and cardiovascular disease (submitted)
3. Marchesini G., Brizi M., Bianchi G. et al. Nonalcoholic fatty liver disease: a feature of the metabolic syndrome. Diabetes 2001;50:1844-50
4. Tilg A, Kaser A. Treatment strategies in nonalcoholic fatty liver disease. Nat Clin Pract Gastroent Hepatol 2005;2:148-55
5. Yokohama S, Yoneda M, Haneda M, et al. Therapeutic efficacy of an angiotensin II receptor antagonist in patients with nonalcoholic steatohepatitis. Hepatology 2004;40:1222-5
6. Neuschwander-Tetri BA, Brunt EM, Wehmeier KR, et al. Improved nonalcoholic steatohepatitis after 48 weeks of treatment with the PPAR-gamma ligand rosiglitazone. Hepatology 2003;38:1008-17
7. Tiikkainen M, Hakkinen AM, Korsheninnikova E, et al. Effects of rosiglitazone and metformin on liver fat content, hepatic insulin resistance, insulin clearance, and gene expression in adipose tissue in patients with type 2 diabetes. Diabetes 2004 ;53:2169-76
8. Ryysy L, Hakkinen AM, Gotto T, et al. Hepatic fat content and insulin action on free fatty acids and glucose metabolism rather than insulin absorption are associated with insulin requirements during insulin therapy in type 2 diabetic patients. Diabetes 2000;49:749-58
9. Steneberg P, Rubins N, Bartoov-Shifman R, et al. The FFA receptor GPR40 links hyperinsulinemia, hepatic steatosis, and impaired glucose homeostasis in mouse. Cell Metab 2005;1:245-58
10. Musso G, Gambino R, Biroli G, et al. Hypoadiponectinemia predicts the severity of hepatic fibrosis and pancreatic β-cell dysfunction in nondiabetic nonobese patients with NASH. Am J Gastroenterol 2005;100:2438-46
11. Diamant M, Lamb HJ, Van de Ree MA, et al. The association between abdominal visceral fat and carotid stiffness is mediated by circulating inflammatory markers in uncomplicated type 2 diabetes. J Clin Endocrinol Metab 2005;90:1495-501
12. Tushuizen ME et al. Liverfat is elevated in the metabolic syndrome and DM2 and Associated with postprandial dysmetabolism. Abstract (submitted ADA 2006)

Appendix 6
Sub-Study 2
Fatty acid partitioning / muscle and subcutaneous fat biopsies
PRESERVE PROTOCOL

Previous approval by the Medical Ethical Review Committee of Maastricht University/azM for studies in which the same techniques were used as will be in this study:

The Medical Ethical Review Committee of Maastricht University/azM has previously given approval to perform the following studies, in which exactly the same techniques (including adipose tissue and skeletal muscle biopsies) were used as will be in the present sub-study:
Arterio-venous differences across the forearm with/without stable isotope infusions

- **MEC 04-149.3**
- **MEC 03-093.3**
- **MEC 02-035.3**
- **MEC 01-104.3**
- **MEC 98.241.5 (amendment MEC 98.241.11)**
- **MEC 02-128.7**
- **MEC 99-060.1**

BACKGROUND
PPARγ agonists or thiazolidinedione(TZD; e.g. rosiglitazone) or blockade of the renin-angiotensin sysem (AT1 receptor blockers like valsartan) may improve insulin sensitivity by promoting the recruitment and differentiation of adipocytes, thereby decreasing fatty acid flux to non-adipose tissues and reducing ectopic fat storage (decreasing lipid accumulation in skeletal muscle, liver and/or pancreas).

HYPOTHESES
1. The insulin sensitizing effect of TZD may be explained by decreasing the lipid flux from adipose tissue to non-adipose tissues, thereby decreasing ectopic fat storage.
2. Most of the vascular and metabolic effects of angiotensin II are mediated by the angiotensin II (Ang II)-type 1 (AT1) receptor.[1,2] The beneficial effect of AT1 receptor blockers (ARB) may be mediated by **a)** an increased adipose tissue and skeletal muscle blood flow, thereby affecting postprandial glucose and triglyceride disposal, **b)** an increase of the lipid buffering capacity of adipose tissue thereby decreasing lipid supply to non-adipose tissues.

OBJECTIVES
To compare
   a) skeletal muscle and adipose tissue blood flow
   b) substrate metabolism and fatty acid partitioning between adipose tissue and skeletal muscle
   c) fat cell size (as a measure of adipocyte differentiation)

before and after 52 weeks of rosiglitazone (RSG), valsartan (VAL) or placebo (in a 2 x 2 factorial design) in the PRESERVE population (i.e. prediabetic subjects, with IFG, IGT and a family history of type 2 diabetes).

DESIGN AND METHODS
Stable isotope methodology has been developed to trace fat partitioning within the body during the postprandial state.[3] This methodology in combination with arterio-venous balance techniques across muscle offers the unique possibility to study the effects on fatty acid partitioning from either endogenous or exogenous fat sources *in vivo* in man. Thus, this methodology will provide more insight in the underlying mechanisms by which the PPARγ agonist rosiglitazone and the ARB valsartan may modulate fatty acid metabolism, ectopic fat accumulation and insulin sensitivity *in vivo* in man in relevant populations (ie subjects at high risk for the development of type 2 diabetes mellitus and cardiovascular disease).

Before and 52 weeks after pharmacotherapy with either valsartan, rosiglitazone, valsartan and rosiglitazone, or placebo, subjects from the PRESERVE population (12 in each arm) will complete a stable isotope infusion test (‘fatty acid partitioning test’ - see Appendix 1 - the test is explained below).

Number of subjects: Sample size will be 12 subjects per group, allowing for a drop-out rate of 20%. In previous studies, a number of 10 subjects per group has been shown to be sufficient to detect significant differences in fatty acid metabolism using stable isotope methodology.

STABLE ISOTOPE INFUSION TEST

To separate between the contribution of exogenous and endogenous fatty acid to whole body lipolysis and skeletal muscle fatty acid handling two stable isotope tracers of fatty acids will be used:
1) [^{2}H]-palmitate tracer, which will be continuously intravenously infused over the whole study period (from time -120 to 240 min)
2) [U-^{13}C]-palmitate tracer, which will be orally ingested with a high fat mixed meal (ingested at t=0)

Rate of appearance of fatty acids (FA), free FA uptake/release across forearm muscle, and triglyceride-derived FA uptake across muscle (and the contribution of endogenous/exogenous fatty acids to this process) will be determined by combining arterio-venous blood sampling across forearm muscle and measurement of forearm blood flow (see 'blood flow measurements' below). Additionally, plasma glucose, lactate, glycerol and insulin concentrations will be determined.
During this test energy expenditure and substrate oxidation will be determined by whole-body indirect calorimetry.

Blood flow measurements

Forearm blood flow. Forearm blood flow will be measured by venous occlusion plethysmography using mercury-in-silastic strain gauges applied to the widest part of the forearm.[4] During measurement periods, the hand circulation will be occluded by rapid inflation of a pediatric sphygmomanometer cuff, placed around the wrist, to a pressure of 200 mmHg. In this way, forearm blood flow can be assessed without interference of the hand circulation. The cuff placed around the upper arm, will be automatically inflated (and deflated) to 40 mmHg to achieve venous occlusion and obtain plethysmographic recordings.
Adipose tissue blood flow. Adipose tissue blood flow will be measured using the ^{133}Xe washout technique.[5] A dose of 2 MBq of the radioactive ^{133}Xe dissolved in sterile saline (9 g/L NaCl) will be injected para-umbilically into the adipose tissue, approximately 10 mm deep, using an insulin-injection syringe with a fine needle (0.36 mm external diameter). The amount of radioactivity used is extremely small and is equivalent to about one-fiftieth of a chest X-ray. After injection the needle will be withdrawn slowly to avoid passage of ^{133}Xe along the injection track. A CsI crystal detector (Oakfield Instruments, Eynsham, UK) will be placed over the exact site of injection and taped firmly in place to monitor the residual radioactivity in the adipose tissue. This γ-counter probe collects continuous 20 s readings.[6]

STUDY DAY

Subjects will arrive at the laboratory by car or bus after an overnight fast. First, a DEXA scan will be performed to determine body composition. Therafter, muscle biopsy will be taken from the *m. vastus lateralis* under local anesthesia by means of a Bergström needle with suction.
Subsequently, three cannulas will be inserted before the start of the experiment. Arterialized venous blood will be obtained through a cannula inserted retrogradely into a superficial dorsal hand vein. The hand will be warmed in a hot-box, which is maintained at 60ºC.[7] In the same arm, a second cannula will be inserted in a forearm antecubital vein for the stable isotope infusion. In the contralateral arm, a third catheter will be introduced retrogradely in an antecubital vein of the forearm for sampling of deep venous blood. The subjects will be in supine position for the duration of the study.
After an arterialized blood sample has been drawn to determine background isotopic enrichment, a ^{2}H-palmitate infusion (0.04 µmol/kg bw/min (palmitate bound to albumin) will be administered intravenously during the whole study period (start at -120 minutes).
Arterio-venous blood sampling and blood flow measurements will be done at time points t= -30, -15 and -5 min during the baseline period. Blood samples will be taken simultaneously from the arterialized vein and the deep forearm vein.
At time t=0, subjects will orally ingest 100 mg of uniformly labeled U-13C-palmitate, emulsified with a 3.0 MJ liquid testmeal, consisting of 62,3 en% fat (40 en% SF, 40,4 en% MUFA, 19,6 en% PUFA), 32,6 en% CHO and 5,1 en% proteins containing 13C tracer (the meal will consist of rice krispies, milk and chocolate lipid emulsion).
Arterio-venous blood sampling and blood flow measurements will occur at time t= -30, -20, -10, 30, 60, 90, 120, 180 and 240 min as described above. At the end of the study, a second muscle biopsy will be taken

from the *m. vastus lateralis*. This muscle biopsy will be collected through the same incision as the muscle biopsy at baseline.

$^2H_2$-palmitate infusion
and
continuous adipose tissue blood flow measurement (^{133}Xe wash-out)

-120	-30	-20	-10	0	30	60	90	120	180	240
*	*	*	*		*	*	*	*	*	*
	#	#	#		#	#	#	#	#	#
	§	§	§		§	§	§	§	§	§
M				Meal						M

* = arterialized and deep venous blood sampling
\# = plethysmography (forearm blood flow measurement)
§ = ventilated hood (indirect calorimetry)
M = muscle biopsy
Meal = meal ingestion, containing U-^{13}C-palmitaat

Analyses Stable Isotope test
^{2}H and ^{13}C enrichment in plasma TAG and FFA will be analysed by means of GC-MS (^{2}H) and GC-cIRMS (^{13}C). Insulin will be analyzed by radioimmunoassay, and glucose, lactate and glycerol and triglyceride concentrations will be determined by standard enzymatic techniques on an automated spectrophotometer. Oxygen saturation of hemoglobin will be determined on a blood gas analyzer.

Adipose tissue biopsy
During visit 2 (week -2) and 12 (week 52), an adipose tissue biopsy will be collected: a small amount (approximately 1g) of abdominal subcutaneous adipose tissue will be collected under local anesthesia using needle biopsy (with the needle connected to a vacuum syringe) to examine the effect of rosiglitazone and valsartan treatment on fat cell size and inflammation; see 'Hypotheses II, b and f' on page 6).

Risks of this sub-study
Venapunctures can occasionaly cause a local hematoma or bruise to occur. Some participants report pain during venapuncture.
Due to the local anesthesia, the fat and muscle biopsy is as good as painless. Some participants, however, do report a sense of pressing pain during muscle biopsy. This tension is very comparable to pain that occurs upon bumping against a table edge. Occasionally both procedures might cause a local hematoma or bruise to occur. To minimize the risk for a hematoma, the muscle biopsy place will be taped with an elastic adhesive compression bandage, and the fat biopsy place will be compressed for approximately 5 minutes after biopsy. The place of incision will leave a small scar (~ 3 mm). To promote good wound healing, the incision will be sealed with sterile steristrips and a waterproof band-aid.

References
1. Goossens GH, Blaak EE, Saris WH, et al. Angiotensin II-induced effects on adipose and skeletal muscle tissue blood flow and lipolysis in normal-weight and obese subjects. J Clin Endocrinol Metab 2004;89:2690-6
2. Goossens GH, Blaak EE, Van Baak M. Possible involvement of the adipose tissue renin-angiotensin system in the pathophysiology of obesity and obesity-related disorders. Obes Rev 2003;4:43-55
3. Blaak EE. Metabolic fluxes in skeletal muscle in relation to obesity and insulin resistance. Best Pract Res Clin Endocrinol Metab 2005;19:391-403
4. Webb DJ. The pharmacology of human blood vessels in vivo. J Vasc Res 1995; 32: 2-15
5. Larsen OA, Lassen NA, Quaade F. Blood flow through human adipose tissue determined with radioactive xenon. Acta Physiol Scand 1966; 66: 337-345

6. Samra JS, Frayn KN, Giddings JA, Clark ML, Macdonald IA. Modification and validation of a commercially available portable detector for measurement of adipose tissue blood flow. Clin Physiol 1995; 15: 241-248
7. Abumrad NN, Rabin D, Diamond MP, Lacy WW. Use of a heated superficial hand vein as an alternative site for the measurement of amino acid concentrations and for the study of glucose and alanine kinetics in man. Metabolism 1981; 30: 936-940
8. Frayn KN, Coppack SW, Humphreys SM, Whyte PL. Metabolic characteristics of human adipose tissue in vivo. Clin Sci (Colch) 1989; 76: 509-516
9. Coppack SW, Frayn KN, Humphreys SM, Dhar H, Hockaday TD. Effects of insulin on human adipose tissue metabolism in vivo. Clin Sci (Colch) 1989; 77: 663-670
10. Coppack SW, Frayn KN, Humphreys SM, Whyte PL, Hockaday TD. Arteriovenous differences across human adipose and forearm tissues after overnight fast. Metabolism 1990; 39: 384-390

Appendix 7
Sub-Study 3
Ultrasound assessments of carotid intima media thickness / distensibility
PRESERVE PROTOCOL

Carotid intima media thickness (CIMT) is a well-established indicator of atherosclerosis as well as a generally accepted so-called surrogate cardiovascular disease end-point (refs). Other surrogate markers are carotid interadventitial diameter (IAD) and carotid distensibility (CD).
For the current protocol, vascular measurements will be performed at the VUMC and Hoorn locations only.

CIMT, IAD and DC will be measured at the right common carotid artery by well-trained observers using a high-resolution ultrasonic wall-track system (WTS, PieMedical, Maastricht, NL; AU-5, Esaote, Maastricht, NL) as previously described. The scanner is connected to a personal computer equipped with wall track software that enables measurement of IMT and IAD. From IMT and IAD, LD is calculated as LD= IAD-(2 · IMT) in millimeters. Circumferential wall stress (σ_C) will be calculated as σ_C=PP(LD/IMT) in kilopascals, where PP is carotid pulse pressure (PP) estimated by distension waveform calibration.

Ad c) *CD* indices are determined by arterial ultrasonography, using previously described techniques (Henry 2006) and calculated as follows:
- Distensibility coefficient = $(2\Delta D \cdot D + \Delta D^2) / (\Delta P \cdot D^2)$ in $10^{-3} \cdot kPa^{-1}$
- Compliance coefficient = $\pi \; (2D \cdot \Delta D + \Delta D^2) / (4 \cdot \Delta P)$ in $mm^2 \cdot kPa^{-1}$,
- Young's elastic modulus = D / (IMT · distensibility coefficient) in kPa

where ΔD is distension, D is diameter, and ΔP is local pulse pressure. The distensibility coefficient reflects the arterial elastic properties, whereas the compliance coefficient reflects the arterial buffering capacity and Young's elastic modulus indicates the intrinsic elastic wall properties.

References:

1. Van der Meer IM, Bots ML, Hofman A, et al. Predictive Value of Noninvasive Measures of Atherosclerosis for Incident Myocardial Infarction: The Rotterdam Study. Circulation 2004;109:1089-94
2. Amar J, Ruidavets JB, Chamontin B, et al. Arterial stiffness and cardiovascular risk factors in a population-based study. J Hypertens 2001;19:381-7
3. Henry RM, Kostense PJ, Spijkerman AM, et al. Arterial stiffness increases with deteriorating glucose tolerance status: the Hoorn Study. Circulation 2003;107:2089-95
4. Van Bortel L, Balkestein EJ, van der Heiden-Spek JJ, et al. Non-invasive assessment of local arterial pulse pressure: comparison of applanation tonometry and echo-tracking. J Hypertens 2001;19:1037-44
5. Henry RM, Kostense PJ, Dekker JM, et al. Carotid arterial remodeling: a maladaptive phenomenon in type 2 diabetes but not in impaired glucose metabolism: the Hoorn study. Stroke 2004;35:671-6
6. Diamant M, Lamb HJ, Van de Ree MA, et al. The association between abdominal visceral fat and carotid stiffness is mediated by circulating inflammatory markers in uncomplicated type 2 diabetes. J Clin Endocrinol Metab 2005;90:1495-501
7. O'Rourke MF, Staessen JA, Vlachopoulos C, et al. Clinical applications of arterial stiffness; definitions and reference values. Am J Hypertens 2002;15:426-44

Appendix 8
Sub-Study 3
Microvascular function assessments
PRESERVE PROTOCOL

Background:
Recently, impaired microvascular function was proposed as an important mechanism in the pathogenesis of insulin resistance and the associated cardiovascular risk factors. Microvascular dysfunction in the basal state and during hyperinsulinemia may in part explain the defects in the ability of insulin to increase glucose-uptake in insulin resistant states, including obesity and hypertension. Therapeutic strategies targeting the improvement of microvascular function may also ameliorate insulin resistance and the associated CVD risk factors, most notably hypertension. Alternatively, increasing insulin sensitivity may have beneficial effects on the microcirculation.
Videomicroscopy to measure skin capillary density and capillary recruitment after arterial occlusion and skin endothelium (in)dependent vasodilation assessed by laser Doppler flowmetry together with iontophoresis of acetylcholine and sodium nitroprusside, are among the methods currently used to measure microvascular function in humans. Microvascular function - measured by these methods - strongly associates with insulin sensitivity and blood pressure. In addition, obese subjects and those with DM2 are characterized by microvascular function.
Both study drugs used in the PRESERVE study, i.e. rosiglitazone and valsartan, may have beneficial effects on microvascular function, albeit through different mechanisms. To date, however, there is only few data available in the literature regarding the effects of these drugs on microcirculation and the methods used are invariably different from our techniques.

Objective:
Primary
To assess the changes in microvascular function, measured by videomicroscopy and laser Doppler flowmetry, from baseline after a 52-week treatment with a thiazolidinedione, rosiglitazone (RSG) and an angiotensin-receptor blocker, valsartan (VAL) in patients with IFG and IGT, using a 2 x 2 factorial design within the PRESERVE study.

Secondary
1. To assess the association between treatment-induced changes in microcirculation and insulin resistance (measured during the hyperinsulinemic-euglycemic part of the clamp) and parameters of β-cell function
2. To assess the association between treatment-induced changes in microcirculation and metabolic changes, and alterations in circulating markers of inflammation

For study design, subjects and follow up, please see the PRESERVE study. Microcirculation measurements will be performed at Visit 3 (Week -1) and Visit 12 (Week 52) prior to blood collection.

Methods
Due to the demanding and extensive measurements in the PRESERVE protocol, we will only measure skin capillary density and capillary recruitment after arterial occlusion by video-microscopy. No iontophoresis / laser Doppler flowmetry will be performed. Perfused nailfold capillaries in the dorsal skin of the third finger are visualized by a capillary microscope according to a previously described protocol (1,2). In short, fasted subjects will be measured after 30 min of acclimatization in a quiet, temperature-controlled room in the sitting position. Two separate visual fields of 1 mm^2 will be recorded before and after 4 min of arterial occlusion with a digital cuff, and the images will be stored on videotape for off-line analysis, which consists of counting the number of capillaries at baseline and directly after cuff release. Capillary density is defined as the number of erythrocyte-perfused capillaries per square millimeter of nailfold skin. Percentage capillary recruitment during post-occlusive reactive hyperemia is assessed by dividing the capillary density after during post-occlusive reactive hyperemia by the number of capillaries at baseline.

Sample size
Since these measurements have never been performed before in a study using similar drug interventions in a comparable population, a formal sample size calculation can not be made. However, based on previous

measurements in hypertensive and obese subjects (3,4), we estimate that data obtained from a total number of 80 patients, measured before and after 52 weeks of drug intervention will yield meaningful results.

References

1. Serné EH, et al. Microvascular function relates to insulin resistance and blood pressure in normal subjects. Circulation 1999;99:896-902
2. Serné EH, et al. Capillary recruitment is impaired in essential hypertension and relates to insulin's metabolic and vascular actions. Cardiovasc Res 2001;49:161-8
3. Serné EH, et al. Impaired skin capillary recruitment in essential hypertension is caused by both functional structural capillary rarefaction. Hypertension 2001;38:238-42
4. Serné EH, et al. Direct evidence for insulin-induced capillary recruitment in skin of healthy subjects during physiological hyperinsulinemia. Diabetes 2002;51:1515-22
5. Clark MG, et al. Blood flow and muscle metabolism: a focus on insulin action. AJP 2003;284:E241-E258
6. De Jongh RT, et al. Impaired microvascular function in obesity: implications for obesity-associated microangiopathy, hypertension and insulin resistance. Circulation 2004;109:2529-35
7. Mathieu-Costello O, et al. Regulation of skeletal muscle morphology in DM2 subjects by troglitazone and metformin: relationship to glucose disposal. Metabolism 2003;52:540-6
8. Forst T, et al. Influence of glucose control and improvement of insulin resistance on microvascular blood flow and endothelial function in patients with DM2. Microcirculation 2005;12:543-50.
